# New Monoterpenoid Indole Alkaloids from *Tabernaemontana crassa* Inhibit *β*-Amyloid42 Production and Phospho-Tau (Thr217)

**DOI:** 10.3390/ijms24021487

**Published:** 2023-01-12

**Authors:** Sheng Li, Ling-Ling Han, Ke-Pu Huang, Ye-Han Ma, Ling-Li Guo, Yarong Guo, Xiaoqian Ran, Yong-Gang Yao, Xiao-Jiang Hao, Rongcan Luo, Yu Zhang

**Affiliations:** 1 Key Laboratory of Phytochemistry and Plant Resources in West China, Kunming Institute of Botany, Chinese Academy of Sciences, Kunming 650201, China; 2 Key Laboratory of Animal Models and Human Disease Mechanisms of the Chinese Academy of Sciences & Yunnan Province, and KIZ-CUHK Joint Laboratory of Bioresources and Molecular Research in Common Diseases, Kunming Institute of Zoology, Chinese Academy of Sciences, Kunming 650204, China; 3 School of Life Sciences, Division of Life Sciences and Medicine, University of Science and Technology of China, Hefei 230026, China; 4 Kunming College of Life Science, University of Chinese Academy of Sciences, Kunming 650201, China

**Keywords:** *Tabernaemontana crassa*, monoterpenoid indole alkaloids, Aβ42, phospho-tau (Thr217), Alzheimer’s disease

## Abstract

Eleven monoterpenoid indole alkaloids, including three new ones, tabercrassines A–C (**1**–**3**), were isolated from the seeds of *Tabernaemontana crassa*. Tabercrassine A (**1**) is an ibogan–ibogan-type bisindole alkaloid which is formed by the polymerization of two classic ibogan-type monomers through a C3 unit aliphatic chain. Their structures were established by extensive analysis of HRESIMS, NMR, and ECD spectra. Cellular assays showed that alkaloids **1**–**3** all reduce A*β*42 production and inhibit phospho-tau (Thr217), a new biomarker of Alzheimer’s disease [AD] associated with BACE1-, NCSTN-, GSK3β-, and CDK5-mediated pathways, suggesting these alkaloids’ potential against AD.

## 1. Introduction

Alzheimer’s disease (AD), the most common type of dementia, accounts for up to 70% of neurodegenerative diseases [1,2,3]. It is now one of the most common fatal diseases, and over 50 million individuals worldwide suffer from AD [4]. The main pathological hallmarks of AD are senile plaques and neurofibrillary tangles (NFTs) composed of amyloid beta (A*β*) and over-phosphorylated tau protein, respectively [3,5,6,7]. However, as AD is a heterogeneous, polygenic, and complex disease, there are no efficacious disease-modifying therapeutics to date [7,8]. Hence, the discovery of a diversification of AD-druggable poly-targets or therapeutic drug combination strategies might provide new drug discovery avenues [8]. Among these avenues, inhibitors of amyloid precursor protein (APP) protease, β-site cleaving enzyme 1 (BACE1), and cyclin-dependent kinase 5 (CDK5) contributing to tau phosphorylation have been suggested as appealing drug targets for AD [9,10,11,12]. Previous studies have shown that natural products exhibited biological activities against AD by inhibiting cyclin-dependent kinase 5 and tau phosphorylation and reducing A*β*42 and A*β*40 production toward the nonamyloidogenic pathway [13,14].

Monoterpenoid indole alkaloids (MIAs) are a category of natural products that are attractive due to their intriguing skeletons and promising bioactivities [15,16,17]. Among MIAs, reserpine, vincristine and its derivatives are outstanding representatives [18,19]. The genus *Tabernaemontana* (Apocynaceae family) comprises 99 species worldwide, some of which have been widely used as folk medicine for the treatment of hypertension, snake poisoning, and rheumatalgia [20]. Interestingly, ibogaine is the most abundant ibogan-type MIA in the genus *Tabernaemontana*, and has been used as a psychopharmacological sacrament in the Bwiti religion (West Africa) for several centuries [21]. In recent years, various classes of MIAs have been isolated and identified from this genus and some of them possessed novel skeletons and intriguing biological activities [22,23,24,25]. Previously, we found that kopoffines A–C, which are MIA dimers, showed significant inhibition against cyclin-dependent kinase 5 and decreased the protein levels of phospho-tau (pTau217 and pTau396) without influencing A*β* production [13].

**Figure 1:**
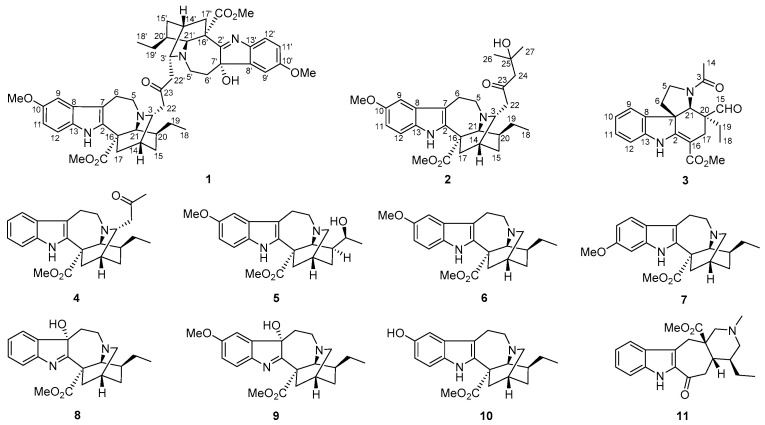
Molecular structures of **1**–**11**.

In the continuous search for structurally intriguing MIAs and anti-AD lead compounds, three new ones—tabercrassines A–C (**1**–**3**)—along with eight known ones were isolated and identified from the seeds of *T. crassa*. Alkaloid **1** was a dimer, which was assembled by two ibogan-type monomers through a C3 unit aliphatic chain, while Alkaloid **2** was also an ibogan-type MIA characterized by a C6 unit aliphatic chain. Alkaloid **3** represents a rare aspidosperma-type MIA with the fracture at the C-14 and C-15 positions. The eight known alkaloids were identified as 3-(2′-oxopropyl)-coronaridine (**4**) [26], voacristine (**5**) [27], voacangine (**6**) [26], isovoacangina (**7**) [28], coronaridine hydroxyindolenine (**8**) [29], 7α-voacangine hydroxyindolenine (**9**) [30], 10-hydroxycoronaridine (**10**) [31], and ervatamine (**11**) [32] (Figure 1). Alkaloids **1**–**3** showed potential amyloid *β* and tau protein-targeted inhibitory effects in AD cellular models. Herein, we reported the isolation, structural elucidation, and biological activities of the new isolates.

## 2. Results and Discussion

### 2.1. Characterization of the New Isolates

**Figure 2:**
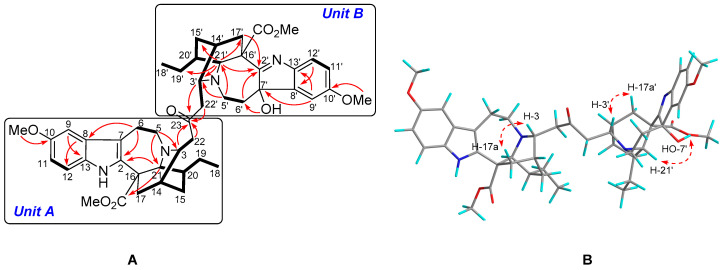
Notes: ^1^H−^1^H COSY ((**A**): bold lines), selected HMBC ((**A**): →), and ROESY ((**B**): ↔) correlations of **1**.

Tabercrassine A (**1**) was isolated as a white powder. The molecular formula C_47_H_58_N_4_O_8_, which indicated 21 indices of hydrogen deficiency, was obtained through ^13^C NMR data and HRESIMS analysis at *m*/*z* 807.4333 [M + H]^+^ (calcd. 807.4327) (Figures S2 and S7). The UV absorption bands at 229 and 285 nm suggested the presence of an indole chromophore [33], while the IR absorption bands at 3441, 1729, and 1625 cm^−1^ were indicative of the presence of amino, ester, and aromatic functionalities, respectively (Figure S8). The ^1^H NMR spectrum of **1** contains two typical ABX proton coupling patterns (δ_H_ 6.93, d, J = 2.5 Hz, H-9; δ_H_ 6.68, dd, J = 8.5, 2.5 Hz, H-11; δ_H_ 7.16, d, J = 8.5 Hz, H-12; δ_H_ 6.86, d, J = 2.5 Hz, H-9′; δ_H_ 6.81, dd, J = 8.5, 2.5 Hz, H-11′; δ_H_ 7.22, d, J = 8.5 Hz, H-12′), indicated by two indole rings substituted at C-10 (δ_C_ 154.8) and C-10′ (δ_C_ 159.9) (Figure S1). Moreover, two broad singlets (δ_H_ 9.26, 4.53) were the characteristic resonances for NH and OH groups, respectively. The ^13^C NMR data (Table 1) showed 47 carbon atoms comprising 6 methyls, 12 methylenes, 14 methines, and 15 non-protonated carbons. All these signals suggested **1** to be an ibogan–ibogan-type bisindole alkaloid. Direct comparison of its NMR data with voacangine and 7α-voacangine hydroxyindolenine suggested **1** to be formed by the polymerization of the two classic ibogan-type monomers [26,30]. A striking difference was the presence of an additional C3 unit (δ_H_ 2.51, H-22a; δ_H_ 2.72, H-22b; δ_H_ 2.50, H-22′a; δ_H_ 2.68, H-22′b; δ_C_ 46.9, C-22; δ_C_ 210.4, C-23; δ_C_ 47.0, C-22′) in **1**, which might be the key linkage between the two ibogan-type monomers. Furthermore, the two nitrogenated methines (δ_H_ 3.28, δ_C_ 56.4; δ_H_ 3.23, δ_C_ 52.5) were also observed simultaneously. The crucial HMBC cross-peaks of H-3 (δ_H_ 3.28), H-22a, H-3′ (δ_H_ 3.23), and H-22′a to C-23 indicated that the non-protonated carbon resonating at δ_C_ 210.4 was located at C-23. The ^1^H-^1^H COSY correlation of H-3 and H-22a, and of H-3′ and H-22′a established the linkage of a C3 unit aliphatic chain between C-3 (δ_C_ 56.4) and C-3′ (δ_C_ 52.5). Further analysis of the 2D NMR (HSQC, HMBC, ^1^H–^1^H COSY) spectra finally confirmed the planar structure of **1** (Figure 2A and Figures S3–S5).

The relative configuration of **1** was same as voacangine and tabervarine A, based on their identical ROESY correlations [26,34]. The ROESY cross-peaks of H-3 with H-17a (δ_H_ 1.85), and of H-3′ with H-17a′ (δ_H_ 2.35) indicated the β-orientations of H-3 and H-3′ in units A and B. Meanwhile, the ROESY correlation of 7′-OH (δ_H_ 4.53) with H-21′ (δ_H_ 3.96) indicated that 7′-OH took an a-orientation (Figure 2B and Figure S6). The ECD spectrum of **1** showed Cotton effects at 212 nm (−14.4) and 261 nm (+30.2) (Figure S9) which possessed a great similarity with those of voacangine and tabervarine A, respectively [26,34], thereby establishing the absolute configuration of **1** as 3*R*,14*R*,16*S*,20*S*,21*S*,3′*R*,7′*S*,14′*R*,16′*S*,20′*S*,21′*S*.

**Figure 3:**
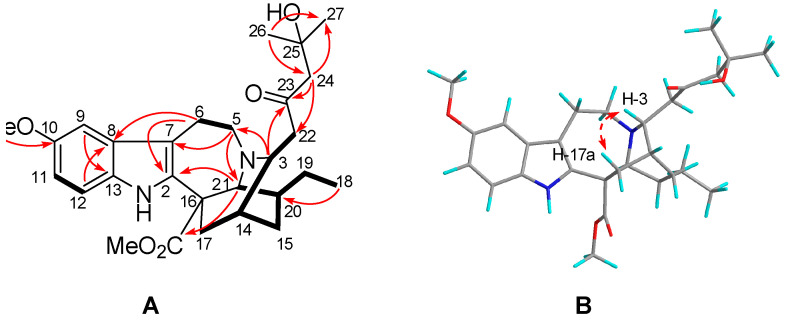
Notes: ^1^H−^1^H COSY ((**A**): bold lines), selected HMBC ((**A**): →), and ROESY ((**B**): ↔) correlations of **2**.

Tabercrassine B (**2**) was obtained as a white powder with a specific rotation of ${\left[\alpha \right]}_{D}^{24}$ −9 (*c* 0.05, MeOH); The molecular formula of C_28_H_38_N_2_O_5_ was established by its HRESIMS data at 483.2852 [M + H]^+^ (calcd for [M + H]^+^ 483.2853) (Figure S16), which indicated 11 indices of hydrogen deficiency. The IR spectrum showed absorption bands at 3400, 1726, and 1626 cm^−1^ (Figure S17), which corresponded to amino, carbonyl, and aromatic functionalities. Analysis of the NMR data (Table 2, Figures S10 and S11) suggested that the structure of **2** shared the same basic skeleton with that of voacangine [26], except for the presence of a C6 unit aliphatic chain group (*δ*_H_ 2.64, H-22a; *δ*_H_ 2.80, H-22b; *δ*_H_ 2.58, H-24a; *δ*_H_ 3.78, H-24b; *δ*_H_ 1.16, H-26; *δ*_H_ 1.15, H-27; *δ*_C_ 211.9, C-23; *δ*_C_ 48.1, C-22; *δ*_C_ 55.6, C-24; *δ*_C_ 69.9, C-25; *δ*_C_ 29.9, C-26; *δ*_C_ 29.8, C-27). The signals are attributed to one downfield non-protonated carbon, two methylenes, one ketone carbonyl, and two methyl groups in a C6 unit, respectively. The presence of ^1^H-^1^H COSY cross-peaks of H-3 (*δ*_H_ 3.37) and H-22a and the HMBC correlations from H-3, H-22b, and H-24a to C-23, as well as H-3 to C-5 (*δ*_C_ 52.4), established that the C6 unit aliphatic chain was located at C-3. Further analysis of the 2D NMR (HSQC, HMBC, and ^1^H-^1^H COSY) spectra determined the planar structure of **2** (Figure 3A and Figures S12–S14).

**Figure 4:**
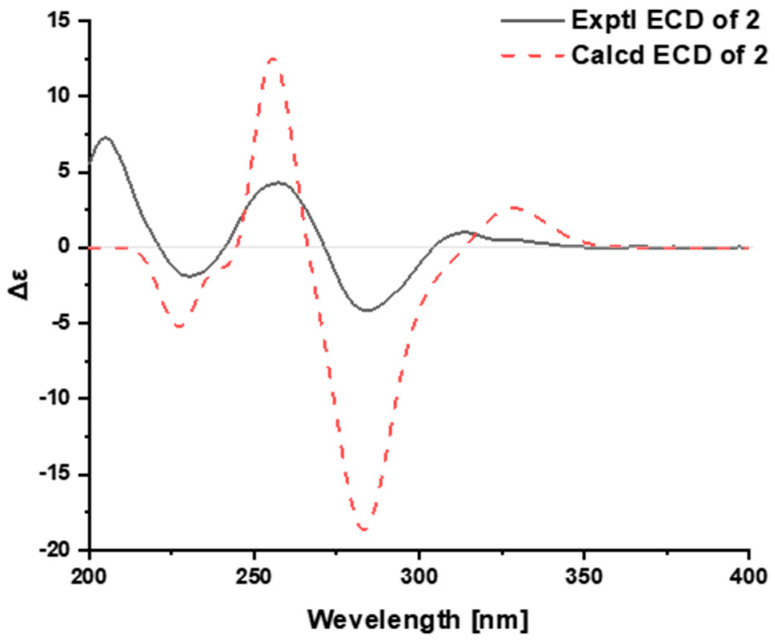
Calculated and experimental ECD of **2**.

The relative configuration of **2** was deduced from the analysis of its ROESY spectrum (Figure 3B), which was identical with that of voacangine. The ROESY correlation of H-3 with H-17a (*δ*_H_ 1.90) indicated that both protons were co-facial and were assigned arbitrarily as *β*-oriented (Figure S15). Finally, time-dependent density functional theory (TDDFT) ECD calculation was applied to clarify the absolute configuration of **2**. The calculated ECD data of (3*R*,14*R*,16*S*,20*S*,21*S*)-**2** matched well with the experimental data (Figure 4, Figures S18 and S28), confirming the absolute configuration of **2** as shown in Figure 1.

**Figure 5:**
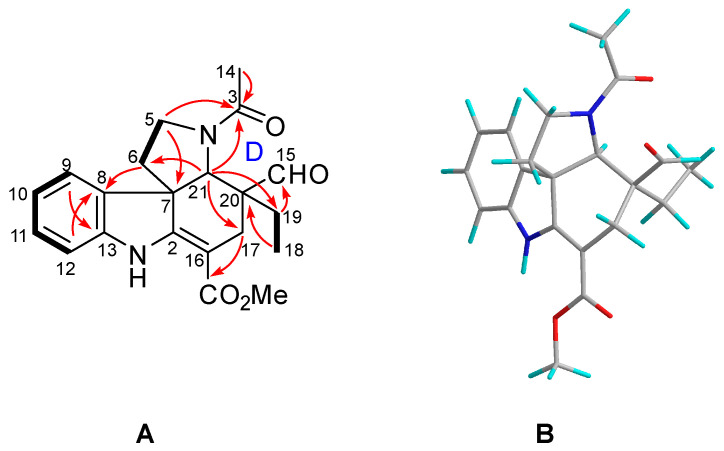
Notes: ^1^H−^1^H COSY ((**A**): bold lines), selected HMBC ((**A**): →), and ROESY ((**B**): ↔) correlations of **3**.

Tabercrassine C (**3**) was separated as a colorless oily substance, with ${\left[\alpha \right]}_{D}^{25}$**-**37 (*c* 0.07, MeOH), whose molecular formula was deduced as C_21_H_24_N_2_O_4_ based on the HRESIMS ion at *m*/*z* 369.1816 [M + H]^+^ (calcd 369.1809) (Figure S25), corresponding to 11 degrees of unsaturation. The IR spectrum showed bands at 3393, 1719, and 1623 cm^−1^ due to NH, aldehyde, and amide functions, respectively (Figure S26). The ^13^C NMR spectrum (Table 2) showed a total of 21 separate carbon resonances, which were classified as three methyls, four methylenes, five methines, and nine non-protonated carbons. Detailed analysis of its NMR data demonstrated that **3** was essentially similar to the aspidosperma-type alkaloid jerantiphylline A [35], indicating both alkaloids had the same basic carbon skeleton. The striking differences were the observation of chemical shifts of C-9 (Δ*δ* +13.6 ppm), C-10 (Δ*δ* −18.2 ppm), C-11 (Δ*δ* −17.5 ppm), and C-13 (Δ*δ* +16.3 ppm) in **3**, which were attributed to the absence of methoxy and hydroxyl moieties in the indole ring of **3**. The ^1^H-^1^H COSY correlation of H-10 (*δ*_H_ 6.87) and H-11 (*δ*_H_ 7.19), and the key HMBC correlations of H-10 with C-8 (*δ*_C_ 136.7) and C-12 (*δ*_C_ 110.9), and of H-11 with C-9 (*δ*_C_ 122.4) and C-13 (*δ*_C_ 144.7), confirmed the above elucidation (Figure 5A). The 2D NMR spectra (Figures S19–S24, Supporting Information) confirmed that the other partial structures of the molecule were the same as jerantiphylline A. It is worth noting that tabercrassine C (**3**) represents the second example of a ring-D-*seco*-tabersonine alkaloid with the fracture at the C-14 and C-15 positions. This ring-opened alkaloid might be originated from a 3-oxotabersonine derivative such as melosine C [36], via a retro-aldol reaction.

**Figure 6:**
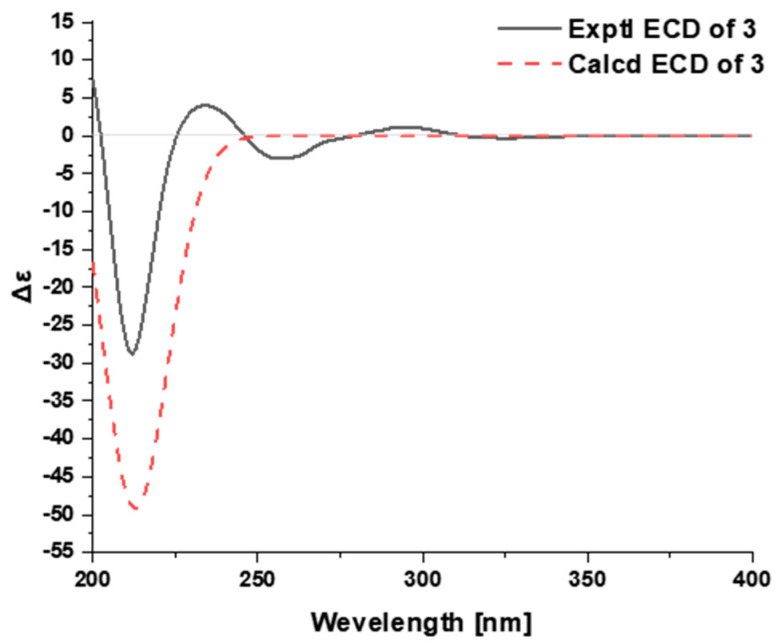
Calculated and experimental ECD of **3**.

The relative configuration of **3** was deduced from the analysis of its ROESY spectrum (Figure 5B and Figure S24). The observed ROESY correlations of H-21 (*δ*_H_ 4.05) and H-18 (*δ*_H_ 0.73), and of H-21 and H-19b (*δ*_H_ 1.81), indicated that the ring-D-*seco*-tabersonine alkaloid had a relative configuration that was identical to that of jerantiphylline A. In an attempt to assign the absolute configuration of **3**, time-dependent density functional theory (TDDFT) ECD calculation was performed. The matched experimental and calculated ECD spectra finally confirmed the absolute configuration of **3** (Figure 6, Figures S27 and S28).

### 2.2. Biological Activity of the New Isolates

**Figure 7:**
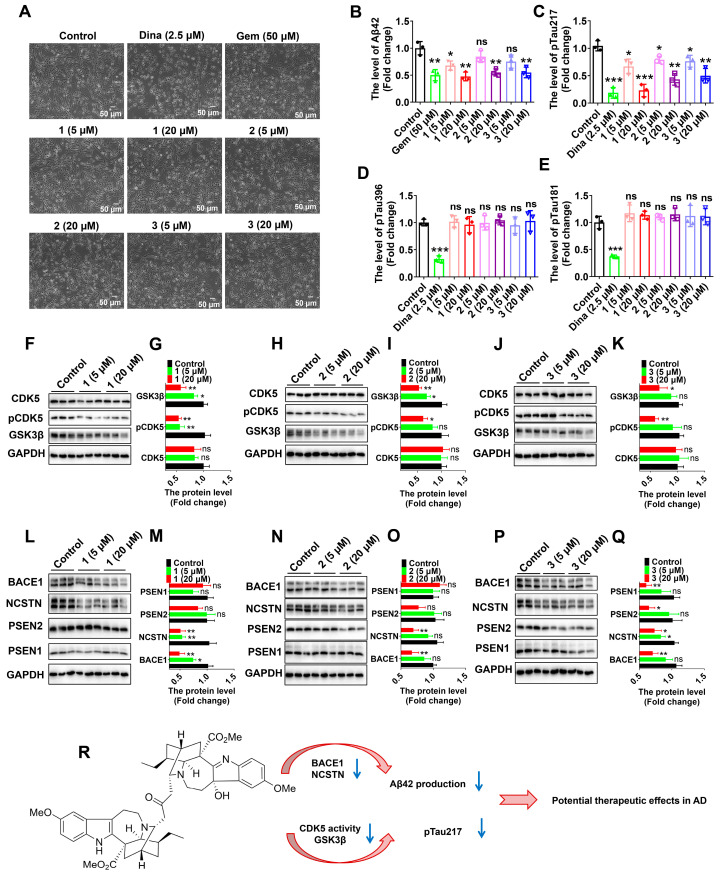
Results of biological activity assays. (**A**) The morphology of the U251-APP cells treated with or without compounds (5 μM or 20 μM), gemfibrozil (Gem, 50 μM, a positive control), or Dinacilib (Dina, 2.5 μM, a positive control) for 24 h. (**B**) Level of extracellular Aβ42 in the culture medium of U251-APP cells treated with compounds, Gem, or DMSO (control), determined by ELISA. (**C**–**E**) Levels of pTau217, pTau396 and pTau181 in the U251-APP cells treated with compounds, Dina, or DMSO (control) determined by ELISA. (**F**–**K**) Western blot assays showing the protein levels of CDK5, pCDK5, and GSK3*β* in the U251-APP cells treated with or without compounds. A representative Western blot result (**F**,**H**,**J**) and quantification of protein levels (**G**,**I**,**K**) based on three independent experiments. (**L**–**Q**) Western blot assays showing the protein levels of BACE1, NCSTN, PSEN2, and PSEN1 in the U251-APP cells treated with or without compounds. A representative Western blot result (**L**,**N**,**P**) and quantification of protein levels (**M**,**O**,**Q**) based on three independent experiments. (**R**) A proposed potential role of **1** against AD by downregulating BACE1, NCSTN, CDK5, and GSK3*β*-mediated pathways, resulting in A*β*42 reduction and decreased pTau217. Data are presented as the means ± SD; ns, not significant; ***, *p* < 0.001; **, *p* < 0.01; and *, *p* < 0.05; one-way ANOVA with Bonferroni’s *post hoc* test.

The cytotoxic activities of the new alkaloids (**1**–**3**) were evaluated against four human cancer cell lines, HepG-2 (liver cancer), CNE-2 (nasopharyngeal carcinoma), HCT-116 (colon cancer), and MDA-MB-231 (triple-negative breast cancer), by using the MTT method, according to our previous studies [23]. Unfortunately, all of them were inactive (IC_50_ > 40 μM). Then, we conducted cellular analyses using human glioma U251 cells stably expressing the human APP mutant (APP-p. K670N/M671L) (U251-APP cells), a cellular AD model that was created in our previous studies [37,38,39]. While DMSO (dimethyl sulfoxide) was used as the solvent and the control, gemfibrozil approved by the US Food and Drug Administration, primarily for treating hyperlipidemia [40,41], was used as a positive control in cellular assays, as it could reduce Aβ production [42] and increase A*β* clearance [37,38,39]. At concentrations of 5 μM and 20 μM, alkaloids **1**–**3** showed no apparent toxicity for U251-APP cells (Figure 7A). We measured the levels of A*β*42 species, which play major synaptotoxic roles in AD [43,44,45]. All of the culture supernatants of U251-APP cells treated with alkaloids **1**–**3** showed a significant decrease in levels of A*β*42 (20 μM), as determined by the enzyme-linked immunosorbent assay (ELISA) (Figure 7B). Interestingly, the effect of **1**–**3** on A*β* production was comparable to that of gemfibrozil at a dose of 20 μM, suggesting that alkaloids **1**–**3** possess the property of preventing A*β* production and its downstream consequence. Additionally, we evaluated anti-tau phosphorylation effects of alkaloids **1**–**3**. We used Dinaciclib, a CDK5 selective inhibitor [46] which can inhibit tau phosphorylation [13], as a positive treatment in this assay. The levels of phospho-tau (Thr217, a new biomarker of AD) [47,48,49], phospho-tau (Thr181, pTau181), and phospho-tau (Ser396, pTau396), which play major roles in the formation of NFT [50], were determined by ELISA. Unexpectedly, we found that alkaloids **1**–**3** all significantly decreased the level of pTau217, whereas the levels of pTau396 and pTau181 were not influenced (Figure 7C–E). These results suggest that these alkaloids can inhibit the phosphorylation of tau and its downstream consequence.

We further measured CDK5 and GSK3β, which play important roles in Tau phosphorylation [51]. Western blot analyses showed that alkaloids **1**–**3** significantly decreased the protein level of GSK3β (Figure 7F–K). Moreover, alkaloids **1**–**3** decreased the level of phospho-CDK5 (Tyr15) (pCDK5), an index of CDK5 enzyme activity [13,52,53,54], although the CDK5 protein level was not changed (Figure 7F–K). These results suggest the potential of alkaloids **1**–**3** to inhibit GSK3β and the CDK5-mediated pathway. In addition, we checked the protein levels of BACE1—the first protease that processes APP in the pathway, leading to the production of toxic A*β* and, therefore, playing a key role in the pathogenesis of AD [3,55]—and the components of *γ*-secretase, including NCSTN (nicastrin), γ-secretase subunit), PSEN1 (presenilin 1), and PSEN2 (presenilin 2). Western blot analyses showed that alkaloids **1**–**3** significantly decreased the protein levels of BACE1 and NCSTN, whereas the protein levels of PSEN1 and PSEN2 were not significantly changed (Figure 7L–Q).

In this study, tabercrassines A–C (**1**–**3**), three MIAs with intriguing structures, were isolated and identified from the seeds of *T. crassa*. Moreover, alkaloids **1**–**3** possess potential bioactivity against AD by inhibiting A*β* production and tau phosphorylation at a site of Thr217 in cellular models (Figure 7R), based on three lines of evidence: (1) alkaloids **1**–**3** decrease A*β*42 production in U251-APP cells; (2) alkaloids **1**–**3** decrease the protein levels of BACE1 and NCSTN; (3) alkaloids **1**–**3** inhibit the protein level of GSK3*β* and the activity of CDK5, and then inhibit the level of pTau217. Thus, it would be rewarding to perform further focused studies by testing whether alkaloids **1**–**3** would inhibit the production of A*β*42 and pTau217, thereby improving cognitive functions in AD animal models.

## 3. Materials and Methods

### 3.1. General Experimental Procedures

Optical rotations, HRESIMS, 1D and 2D NMR, ECD, and IR spectra were obtained as described previously [22,23,24,25]. 

### 3.2. Plant Material

The seeds of *T. crassa* were collected in November 2016 from Ghana, Africa, and were identified by Dr. Paul O. Donkor (School of Pharmacy, University of Ghana, Accra, Ghana). The sample specimen (no. 20161116) was deposited at State Key Laboratory of Phytochemistry and Plant Resources in West China, Kunming Institute of Botany, Chinese Academy of Sciences (CAS).

### 3.3. Extraction and Isolation

The powdered seeds of *T. crassa* (687 g) were extracted with MeOH (2 L), under ultrasonic sound, 3 times (2 h each time) at room temperature. The crude extract (48 g) was separated with a silica gel column eluted with petroleum ether/acetone (100:1–0:1) to yield 3 fractions (A–C). Fraction A (11 g) was further purified by reversed-phase chromatography on a C18 column (MeOH/H_2_O, 40:60→100:0, *v*/*v*), separated with a series of silica gel columns eluted with petroleum ether/acetone (50:1–5:1, *v*/*v*), and further purified by a Sephadex LH-20 column (MeOH) to produce voacangine (14.0 mg), 7*α*-voacangine hydroxyindolenine (4.3 mg), and **3** (2.8 mg). Fraction B (15 g) was separated with a silica gel column (CC) using petroleum ether/acetone (15:1–5:1, *v*/*v*) to obtain 3 subfractions (BI–BIII). BI (3 g) was purified by silica gel column (petroleum ether/acetone, 10:1–0:1) and Sephadex LH-20 column (MeOH) to obtain coronaridine hydroxyindolenine (6.7 mg) and 3-(2′-oxopropyl)-coronaridine (28 mg). Subfraction BII (5.3 g) was separated by Sephadex LH-20 (acetone) and followed by semipreparative HPLC with MeCN/H_2_O (68:32, 0.1% Et_2_NH, 4 mL/min) to obtain voacristine (1.4 mg, t*_R_* = 34.5 min), 10-hydroxycoronaridine (8.3 mg, t*_R_* = 46.0 min), and **2** (3.7 mg, t*_R_* = 52.0 min). Fraction C (7.2 g) was chromatographed with a series of silica gel columns (300–400 mesh) and eluted with a gradient of CH_2_Cl_2_/CH_3_OH (20:1–1:1, *v*/*v*) to yield 2 major subfractions (CI-CII); subfraction CI (2.1 g) was purified by a Sephadex LH-20 (MeOH), followed by semipreparative HPLC using a YMC Triart C18 column (10 × 250 mm, 5 μM) with MeCN/H_2_O (25:75, 0.1% Et_2_NH, 4 mL/min) to obtain isovoacangina (3.4 mg, t*_R_* 31.0 min) and **1** (8.1 mg, t*_R_* 37.0 min). Subfraction CII (2.9 g) was separated by a silica gel column eluted with petroleum ether/acetone (8:1–2:1, *v*/*v*) and followed by semipreparative HPLC using a Waters XBridge C18 (10 × 250 mm, 5 µm) column with MeCN/H_2_O (55:45, 0.1% Et_2_NH, 4 mL/min) to produce ervatamine (3.0 mg, t*_R_* 34.5 min).

### 3.4. General Spectra for Structural Characterization

*Tabercrassine A (***1***)*. White powder; ${\left[\alpha \right]}_{D}^{24}$ +44 (*c* 0.05, MeOH); ECD (0.13 M, MeOH) λ_max_ (∆*ε*) 212 (−14.4), 261 (+30.2) nm; IR (KBr) *v*_max_ 3441, 2954, 1729, 1625, 1475, 1220, 1165, 1027 cm^−1^; ^1^H, and ^13^C NMR data (acetone-*d*_6_, 500, and 125 MHz), see Table 1; HRESIMS *m*/*z* 807.4333 [M + H]^+^ (calcd for C_47_H_58_N_4_O_8_, 807.4327).

*Tabercrassine B (***2***).* White powder; ${\left[\alpha \right]}_{D}^{24}$ −9 (*c* 0.05, MeOH); ECD (0.13 M, CH_3_OH) λ_max_ (∆*ε*) 205 (+7.3), 230 (−1.9), 258 (+4.3), 284 (−4.2); IR (KBr) *v*_max_ 3400, 2929, 1726, 1626, 1457, 1380, 1221, 1032 cm^−1^; ^1^H, and ^13^C NMR data (acetone-*d*_6_, 500, and 125 MHz), see Table 2; HRESIMS *m*/*z* 483.2852 [M + H]^+^ (calcd for C_28_H_38_N_2_O_5_, 483.2853).

*Tabercrassine C (***3***).* Colorless, oily; ${\left[\alpha \right]}_{D}^{25}$**-**37 (*c* 0.07, MeOH); ECD (0.14 M, MeOH) λ_max_ (∆*ε*) 212 (−28.8); IR (KBr) *v*_max_ 3393, 2926, 1719, 1623, 1469, 1385, 1187 cm^−1^; ^1^H, and ^13^C NMR data (acetone-*d*_6_, 600, and 150 MHz) see Table 2; HRESIMS *m*/*z* 369.1816 [M + H]^+^ (calcd for C_21_H_24_N_2_O_4_, 369.1809).

### 3.5. Cytotoxic Activity

The cytotoxic activities of the new alkaloids (**1**–**3**) were evaluated against four human cancer cell lines by using the MTT method, according to our previous studies [23].

### 3.6. Cell Culture and Treatment

The U251-APP cells were cultured in Roswell RPMI-1640 medium (HyClone, Logan, UT, USA, C11875500BT) supplemented with 10% FBS (fetal bovine serum) (Gibco-BRL, Waltham, MA, USA, 10099-141) at 37 °C in a humidified atmosphere incubator with 5% CO_2_ and 95% humidity, as described in our previous studies [37,38,39]. Cells were seeded in pre-warmed growth medium in 6-well plates. Dinaciclib (GLPBIO, Montclair, CA, USA, 779353-01-4) and Gemfibrozil (Abcam, Cambridge, UK, ab142883) were used as positive controls. Drugs were applied to the culture medium directly for treatment; 24 h after drug treatment, the cells were harvested for further analysis.

### 3.7. Western Blot Analysis

Western blotting was performed as in our previous studies [14,38,39,56]. In brief, cell lysates of U251-APP cells were prepared using protein lysis buffer (Beyotime Institute of Biotechnology, P0013). The protein concentration was determined by a BCA protein assay kit (Beyotime Institute of Biotechnology, P0012; Shanghai, China). In total, 20 μg of protein was separated by 12% sodium dodecyl sulfate–polyacrylamide gel electrophoresis and transferred to a polyvinylidene difluoride membrane (Bio-Rad, L1620177 Rev D, Hercules, CA, USA). The membrane was soaked with 5% (*w*:*v*) skim milk at RT for 2 h. The membrane was incubated with primary antibodies overnight at 4 °C. The primary antibodies were BACE1 (Cell Signaling Technology, 5606, Danvers, MA, USA), CDK5 (Santa Cruz Biotechnology, sc-6247, Dallas, TX, USA), GSK3*β* (D5C5Z) (Cell Signaling Technology, 12456), GAPDH (glyceraldehyde-3-phosphate dehydrogenase, Proteintech, 60004-1-Ig), NICSTN (Cell Signaling Technology, 5665), PSEN1 (Cell Signaling Technology, 5643), PSEN2 (Cell Signaling Technology, 9979), phospho-CDK5 (Tyr15) (pCDK5) (Absin, abs130996). The membranes were washed 3 times with TBST (Tris-buffered saline (Cell Signaling Technology, 9997) with Tween 20 (0.1%; Sigma, P1379, St. Louis, MO, USA))—each time for 5 min—and subsequently incubated with peroxidase-conjugated anti-mouse (474-1806) or anti-rabbit (474-1516) IgG (1:5000; KPL) at RT for 1 h. The epitope was visualized using an ECL Western blot detection kit (Millipore, WBKLS0500, Burlington, MA, USA). We used ImageJ (National Institutes of Health, Bethesda, MD, USA) to evaluate densitometry of each blot. GAPDH was used as a loading control to measure the densitometry of target protein.

### 3.8. Enzyme Linked Immunosorbent Assay (ELISA) for Aβ42, pTau217, pTau396 and pTau181

The level of A*β*42 in the culture media of U251-APP cells was determined using commercial ELISA kit (Elabscience, E-EL-H0543c; Wuhan, China), as described in our previous study [14,38,39]. The levels of pTau217, pTau396, and pTau181 in cell lysates were determined using the commercial ELISA kit (RUIFAN, RF13027 to detect pTau217; Elabscience, E-EL-H5314c to detect pTau396; FineTest, EH4701 to detect pTau181), according to the manufacturer’s instructions. We normalized the values of the control group with 3 biological replicates and the other treatment groups were compared with the control group.

### 3.9. Statistical Analysis

Data analyses were carried out by using GraphPad Prism 8 (GraphPad Software, Inc., La Jolla, CA, USA) [38,39,57,58]. The results are expressed as means ± SD. Statistical analysis was performed using one-way ANOVA with Bonferroni’s *post hoc* test, and differences of *p* < 0.05 were considered statistically significant. It was considered to be statistically significant if a *p* value < 0.05. *, *p* < 0.05; **, *p* < 0.01; ***, *p* < 0.001.

## 4. Conclusions

In conclusion, three new MIAs, tabercrassines A–C (**1**–**3**), were obtained from the seeds of *T. crassa*. Intriguingly, alkaloids **1** and **2** represent novel alkaloids within the ibogan-type MIAs category, and **1**–**3** possess potential against AD by inhibiting A*β* production and tau phosphorylation in cellular models. Indeed, studies conducted on extracts or pure compounds of the *Tabernaemontana* genus have reported diverse pharmacological activities including anticancer, antimicrobial, antiviral activities, etc. However, few therapeutic agents from the genus *Tabernaemontana* have been reported for the treatment of neurodegenerative diseases. Our findings shed light on natural products that may provide novel therapeutic strategies for modulating AD from multiple aspects. Thus, it would be rewarding to perform further studies testing whether other ibogan-type MIAs possess the potential for treatment of AD, and whether alkaloids **1**–**3** would improve the cognitive function in AD animal models.

## Figures and Tables

**Table 1:** ^1^H and ^13^C NMR data for 1 in acetone-*d*_6_ (δ in ppm, *J* in Hz) ^a^.

No.	*δ*_H_	*δ*_C_	No.	*δ*_H_	*δ*_C_
2		139.2	2′		188.5
3	3.28 (m)	56.4	3′	3.23 (dd, 8.0, 4.0)	52.5
5a	3.16 (t, 6.0)	52.3	5′a	3.00 (ddd, 15.0, 4.0, 2.0)	47.3
5b	3.22 (ddd, 6.0, 4.0, 1.5) ^b^		5′b	3.34 (ddd, 14.0, 11.0, 4.0)	
6a	3.10 (ddd, 18.5, 7.5, 3.5)	22.6	6′a	1.83 (m) 1.83 (m)	35.1
6b	2.89 (ddd, 18.5, 7.5, 3.5)		6′b		
7		110.2	7′		88.6
8		129.9	8′		145.6
9	6.93 (d, 2.5)	101.0	9′	6.86 (d, 2.5)	108.9
10		154.8	10′		159.9
11	6.68 (dd, 8.5, 2.5)	112.1	11′	6.81 (dd, 8.5, 2.5)	114.2
12	7.16 (d, 8.5)	112.2	12′	7.22 (d, 8.5)	121.7
13		132.2	13′		146.2
14	1.61 (m) ^b^	31.5	14′	1.60 (m) ^b^	31.8
15a	1.20 (m)	27.6	15′a	1.14 (m)	27.8
15b	1.54 (m) ^b^		15′b	1.46 (m)	
16		55.7 ^b^	16′		55.7 ^b^
17a	1.85 (ddd, 16.0, 5.0, 2.5)	38.2	17′a	2.35 (ddd, 14.0, 5.0, 3.0)	37.9 ^b^
17b	2.71 (m) ^b^		17′b	2.76 (dd, 14.0, 2.0)	
18	0.82 (t, 7.5) ^b^	12.0 ^b^	18′	0.82 (t, 7.5) ^b^	12.0 ^b^
19a	1.34 (m) 1.53 (m) ^b^	27.5	19′a	1.30 (m) 1.45 (m)	27.3
19b			19′b		
20	1.25 (m) ^b^	38.9	20′	1.26 (m)	37.9 ^b^
21	3.53 (br s)	59.0	21′	3.96 (br s)	58.6
22a	2.51 (dd, 16.0, 2.0)	46.9	22′a	2.50 (br d, 16.0)	47.0
22b	2.72 (m) ^b^		22′b	2.68 (m) ^b^	
23		210.4			
NH	9.26 (s)		OH-7′	4.53 (s)	
OMe-10	3.78 (s)	55.9	OMe-10′	3.79 (s)	56.0
CO_2_Me		175.2	CO_2_Me’		173.7
	3.63 (s)	52.6		3.58 (s)	52.9

^a 1^H and ^13^C NMR were recorded at 500 and 125 MHz, respectively. ^b^ Overlapped, without designating multiplicity.

**Table 2:** ^1^H and ^13^C NMR data of **2** and **3** in acetone-*d*_6_ (*δ* in ppm, *J* in Hz).

No.	2 ^a^		3 ^b^	
	*δ*_H_	*δ*_C_	*δ*_H_	*δ*_C_
2		139.3		161.1
3	3.37 (dd, 8.5, 4.0)	56.3		171.0
5a	3.20 (dd, 8.0, 6.0) 3.23 (dd, 8.0, 5.0)	52.4	3.77 (ddd, 12.0, 9.0, 1.2)	46.5
5b			3.90 (ddd, 12.0, 9.0, 7.8)	
6a	2.93 (dt, 15.0, 6.0)	22.7	1.95 (m)	38.2
6b	3.14 (ddd, 15.0, 8.0, 5.0)		2.55 (ddd, 9.0, 7.8, 1.2) ^c^	
7		110.2		56.0
8		123.0		136.7
9	6.95 (d, 2.5)	101.0	7.10 (d, 7.2)	122.4
10		154.8	6.87 (td, 7.2, 1.2)	121.9
11	6.70 (dd, 8.5, 2.5)	112.1	7.19 (td, 7.2, 1.2)	129.4
12	7.19 (d, 8.5)	112.3	7.09 (d, 7.2)	110.9
13		132.2		144.7
14	1.71 (m)	31.6	2.11 (s)	22.7
15a	1.28 (m)c	27.6		204.5
15b	1.53 (m)			
16		55.8		89.9
17a	1.90 (ddd, 13.5, 5.0, 2.0)	38.3	2.53 (br d, 16.2) ^c^ 2.67 (br d, 16.2)	23.6
17b	2.75 (dd, 14.0, 2.0)			
18	0.88 (t, 7.5)	12.0	0.73 (t, 7.2)	9.0
19a	1.41 (m)	27.7	1.29 (m)	27.9
19b	1.59 (dt, 13.0, 7.5)		1.81 (m)	
20	1.28 (m) ^c^	38.9		55.6
21	3.57 (br s)	59.0	4.05 (br s)	69.5
22a	2.64 (dd,17.0, 8.0)	48.1		
22b	2.80 (br d, 17.0)			
23		211.9		
24a	2.58 (br d, 6.0) 3.78 (br d, 6.0) ^c^	55.6		
24b				
25		69.9		
26	1.16 (s)	29.9		
27	1.15 (s)	29.8		
OMe-10	3.78 (s)	55.9		
CO_2_Me		175.2		168.1
	3.64 (s)	52.7	3.71 (s)	51.0

^a 1^H and ^13^C NMR were recorded at 500 and 125 MHz, respectively. ^b 1^H and ^13^C NMR were recorded at 600 and 150 MHz, respectively. ^c^ Overlapped, without designating multiplicity.

## Data Availability

Additional supporting information may be found in the online version of this article on the publisher’s website.

## Supplementary Materials

The HRESIMS, NMR, ECD, and IR spectra of compounds **1**–**3** can be downloaded at: https://www.mdpi.com/article/10.3390/ijms24021487/s1. References [59,60,61,62] are cited in the Supplementary Materials.

## Abbreviations

AD	Alzheimer’s disease
NFTs	Neurofibrillary tangles
A*β*	Amyloid beta
APP	Amyloid precursor protein
BACE1	*β*-site APP-cleaving enzyme 1
CDK5	Cyclin-dependent kinase 5
FBS	Fetal bovine serum
MIAs	Monoterpenoid indole alkaloids
TDDFT	Time-dependent density functional theory
ELISA	Enzyme-linked immunosorbent assay
NCSTN	Nicastrin
PSEN1	Presenilin 1
PSEN2	Presenilin 2
GSK3β	Glycogen synthase kinases-3β
pCDK5	Phospho-cyclin-dependent kinase 5 (Tyr 15)
pTau217	Phospho-tau (Thr217)
pTau181	Phospho-tau (Thr181)
pTau396	Phospho-tau (Ser396)
GAPDH	Glyceraldehyde-3-phosphate dehydrogenase
TBST	Tris-buffered saline
