# Supplementary material for: New Monoterpenoid Indole Alkaloids from Tabernaemontana crassa Inhibit β-Amyloid42 Production and Phospho-Tau (Thr217)
Source: Int J Mol Sci. 2023 Jan 12;24(2):1487. doi: 10.3390/ijms24021487 (PMC9862887; doi:10.3390/ijms24021487)

Supplementary Materials

New Monoterpenoid Indole Alkaloids from *Tabernaemontana crassa* Inhibit *β*-Amyloid42 Production and Phospho-Tau (Thr217)

Sheng Li [1], Ling-Ling Han [1], Ke-Pu Huang [1], Ye-Han Ma [1], Ling-Li Guo [1], Yarong Guo [2,3], Xiaoqian Ran [2,4], Yong-Gang Yao [2,4], Xiao-Jiang Hao [1], Rongcan Luo [2,4,]* and Yu Zhang [1,]*

[1] Key Laboratory of Phytochemistry and Plant Resources in West China, Kunming Institute of Botany, Chinese Academy of Sciences, Kunming 650201, China

[2] Key Laboratory of Animal Models and Human Disease Mechanisms of the Chinese Academy of Sciences & Yunnan Province, and KIZ-CUHK Joint Laboratory of Bioresources and Molecular Research in Common Diseases, Kunming Institute of Zoology, Chinese Academy of Sciences, Kunming 650204, China

[3] School of Life Sciences, Division of Life Sciences and Medicine, University of Science and Technology of China, Hefei 230026, China

[4] Kunming College of Life Science, University of Chinese Academy of Sciences, Kunming 650201, China

* Correspondence: luorongcan@mail.kiz.ac.cn (R.L.); zhangyu@mail.kib.ac.cn (Y.Z.)

Contents

Extraction and Isolation.
Figure S1. ^{1}H NMR spectrum of tabercrassine A (**1**) in acetone-d_6 (500 MHz).
Figure S2. ^{13}C NMR spectrum of tabercrassine A (**1**) in acetone-d_6 (125 MHz).
Figure S3. HSQC spectrum of tabercrassine A (**1**) in acetone-d_6.
Figure S4. ^{1}H-^{1}H COSY spectrum of tabercrassine A (**1**) in acetone-d_6.
Figure S5. HMBC spectrum of tabercrassine A (**1**) in acetone-d_6.
Figure S6. ROESY spectrum of tabercrassine A (**1**) in acetone-d_6.
Figure S7. HRESIMS spectrum of tabercrassine A (**1**).
Figure S8. IR spectrum of tabercrassine A (**1**).
Figure S9. ECD spectrum of tabercrassine A (**1**) in MeOH.

Figure S10. ^{1}H NMR spectrum of tabercrassine B (**2**) in acetone-d_6 (500 MHz).
Figure S11. ^{13}C NMR spectrum of tabercrassine B (**2**) in acetone-d_6 (125 MHz).
Figure S12. HSQC spectrum of tabercrassine B (**2**) in acetone-d_6.
Figure S13. ^{1}H-^{1}H COSY spectrum of tabercrassine B (**2**) in acetone-d_6.
Figure S14. HMBC spectrum of tabercrassine B (**2**) in acetone-d_6.
Figure S15. ROESY spectrum of tabercrassine B (**2**) in acetone-d_6.
Figure S16. HRESIMS spectrum of tabercrassine B (**2**).
Figure S17. IR spectrum of tabercrassine B (**2**).
Figure S18. ECD spectrum of tabercrassine B (**2**) in MeOH.

Figure S19. ^{1}H NMR spectrum of tabercrassine C (**3**) in acetone-d_6 (600 MHz).
Figure S20. ^{13}C NMR spectrum of tabercrassine C (**3**) in acetone-d_6 (150 MHz).
Figure S21. HSQC spectrum of tabercrassine C (**3**) in acetone-d_6.
Figure S22. ^{1}H-^{1}H COSY spectrum of tabercrassine C (**3**) in acetone-d_6.
Figure S23. HMBC spectrum of tabercrassine C (**3**) in acetone-d_6.
Figure S24. ROESY spectrum of tabercrassine C (**3**) in acetone-d_6.
Figure S25. HRESIMS spectrum of tabercrassine C (**3**).
Figure S26. IR spectrum of tabercrassine C (**3**).
Figure S27. ECD spectrum of tabercrassine C (**3**) in MeOH.
Figure S28. Calculated and experimental ECD of **2** and **3**.
Figure S29. Uncropped images of western blot in Figure 7.

Extraction and Isolation. The powdered seeds of *T. crassa* (687 g) were extracted with MeOH (2 L) under ultrasonic sound three times (2 h each time) at room temperature. The crude extract (48 g) was separated with a silica gel column eluted with petroleum ether-acetone (100:1-0:1) to yield three fractions (A-C). Fraction A (11 g) was further purified by a reversed phase chromatography on a C18 column (MeOH/H_2O, 40:60→100:0, v/v) and separated with a series of silica gel column eluting with petroleum ether/acetone (50:1–5:1, v/v), and further purified by a Sephadex LH-20 column (MeOH) to afford voacangine (14.0 mg), 7α-voacangine hydroxyindolenine (4.3 mg), and **3** (2.8 mg). Fraction B (15 g) was separated with a silica gel column (CC) using petroleum ether/acetone (15:1–5:1, v/v) to give three subfractions (BI-BIII). BI (3 g) was purified by silica gel column (petroleum ether/acetone, 10:1−0:1) and Sephadex LH-20 column (MeOH) to obtain coronaridine hydroxyindolenine (6.7 mg) and 3-(2'-oxopropyl)-coronaridine (28 mg). Subfraction BII (5.3 g) was separated by Sephadex LH-20 (acetone) and followed by semipreparative HPLC with MeCN/H_2O (68:32, 0.1% Et_2NH, 4 ml/min) to obtain voacristine (1.4 mg, t_R = 34.5 min), 10-hydroxycoronaridine (8.3 mg, t_R = 46.0 min), and **2** (3.7 mg, t_R = 52.0 min). Fraction C (7.2 g) was chromatographed with a series of silica gel column (300-400 mesh) and eluted with a gradient of CH_2Cl_2/CH_3OH (20:1–1:1, v/v) to yield two major subfractions (CI-CII), subfraction CI (2.1 g) was purified by a Sephadex LH-20 (MeOH) and further followed by semipreparative HPLC using a YMC Triart C18 column (10 × 250 mm, 5 μm) column with MeCN/H_2O (25:75, 0.1% Et_2NH, 4 ml/min) to obtain isovoacangina (3.4 mg, t_R 31.0 min) and **1** (8.1 mg, t_R 37.0 min). Subfraction CII (2.9 g) was separated by a silica gel column eluted with petroleum ether/acetone (8:1-2:1, v/v) and followed by

semipreparative HPLC using a Waters XBridge C18 (10 × 250 mm, 5 μm) column with MeCN/H_2O (55:45, 0.1% Et_2NH, 4 ml/min) to afford ervatamine (3.0 mg, t_R 34.5 min).

Figure S1. ^{1}H NMR spectrum of tabercrassine A (1) in acetone-d_6 (500 MHz).

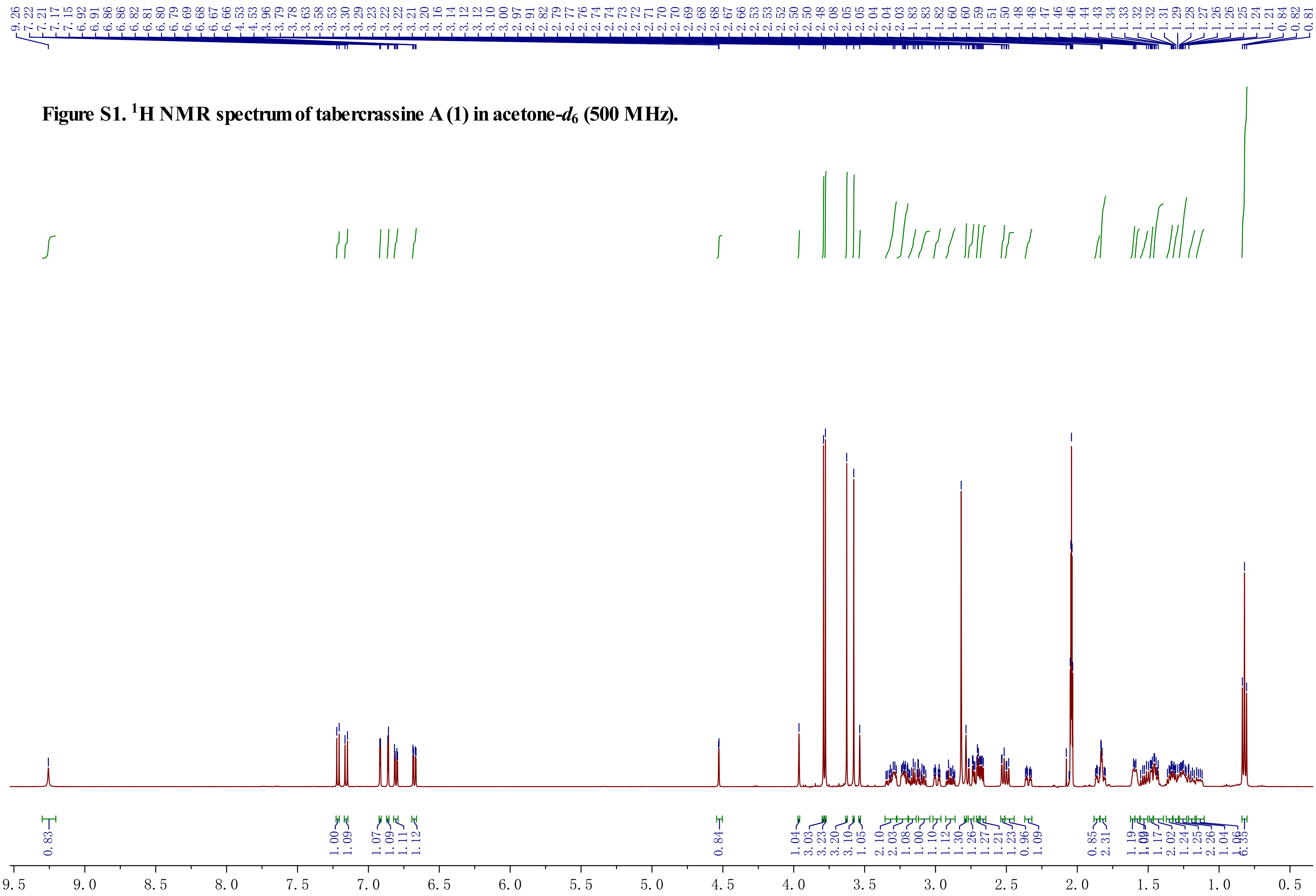

Figure S2. ^{13}C NMR spectrum of tabercrassine A (1) in acetone-d_6 (125 MHz).

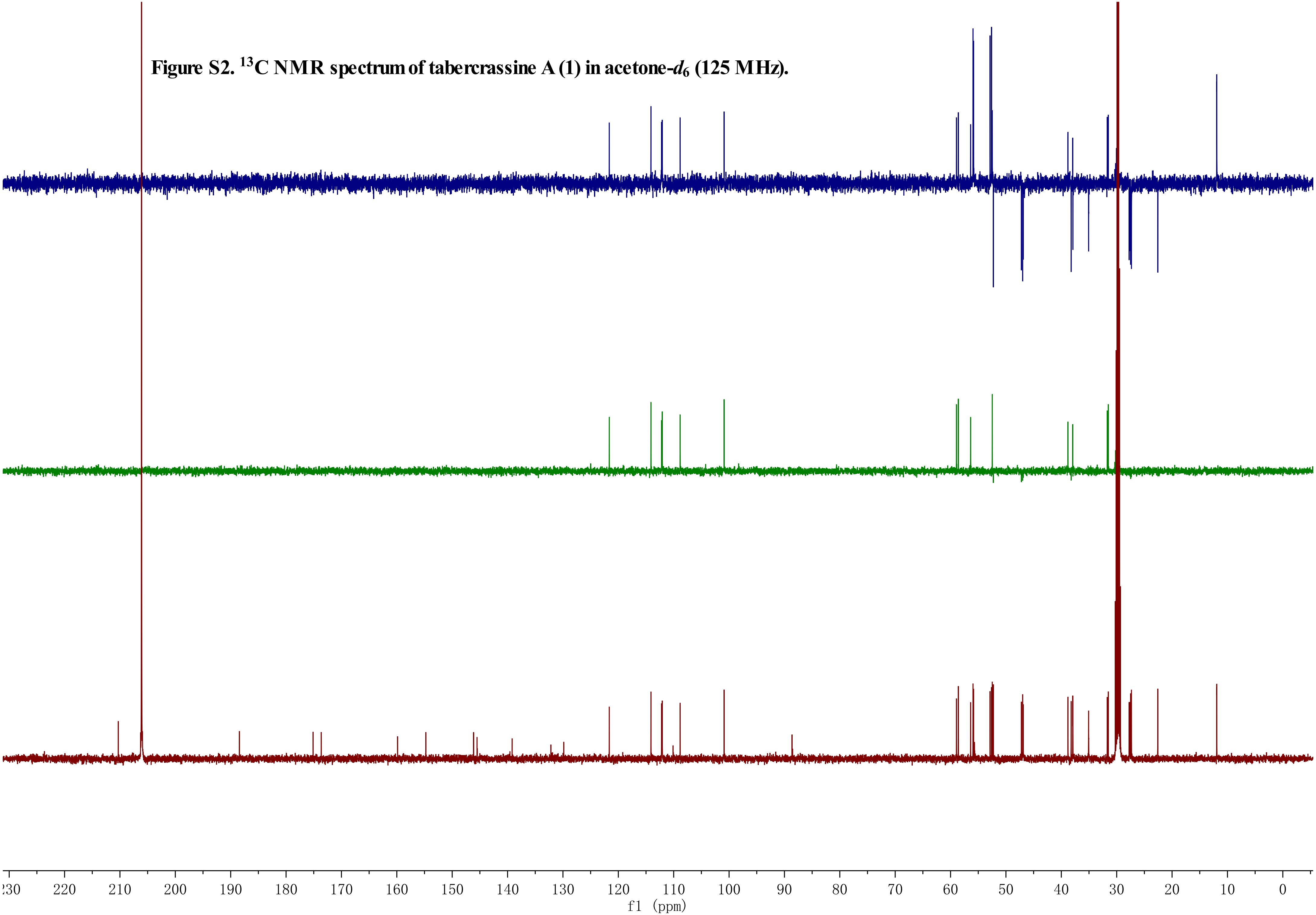

Figure S3. HSQC spectrum of tabercrassine A (1) in acetone-d_6.

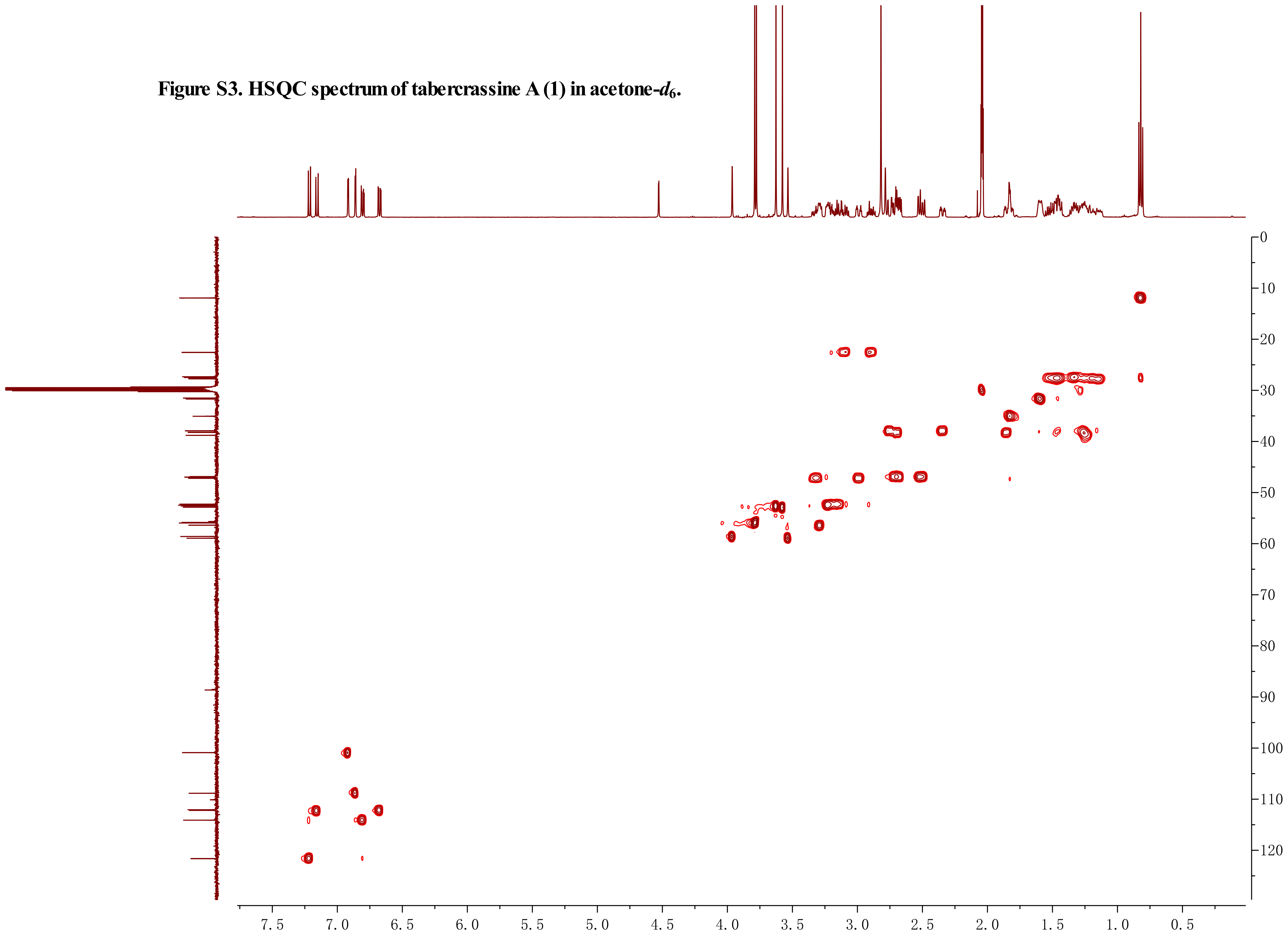

Figure S4. ^{1}H-^{1}H COSY spectrum of tabercrassine A (1) in acetone-d_6.

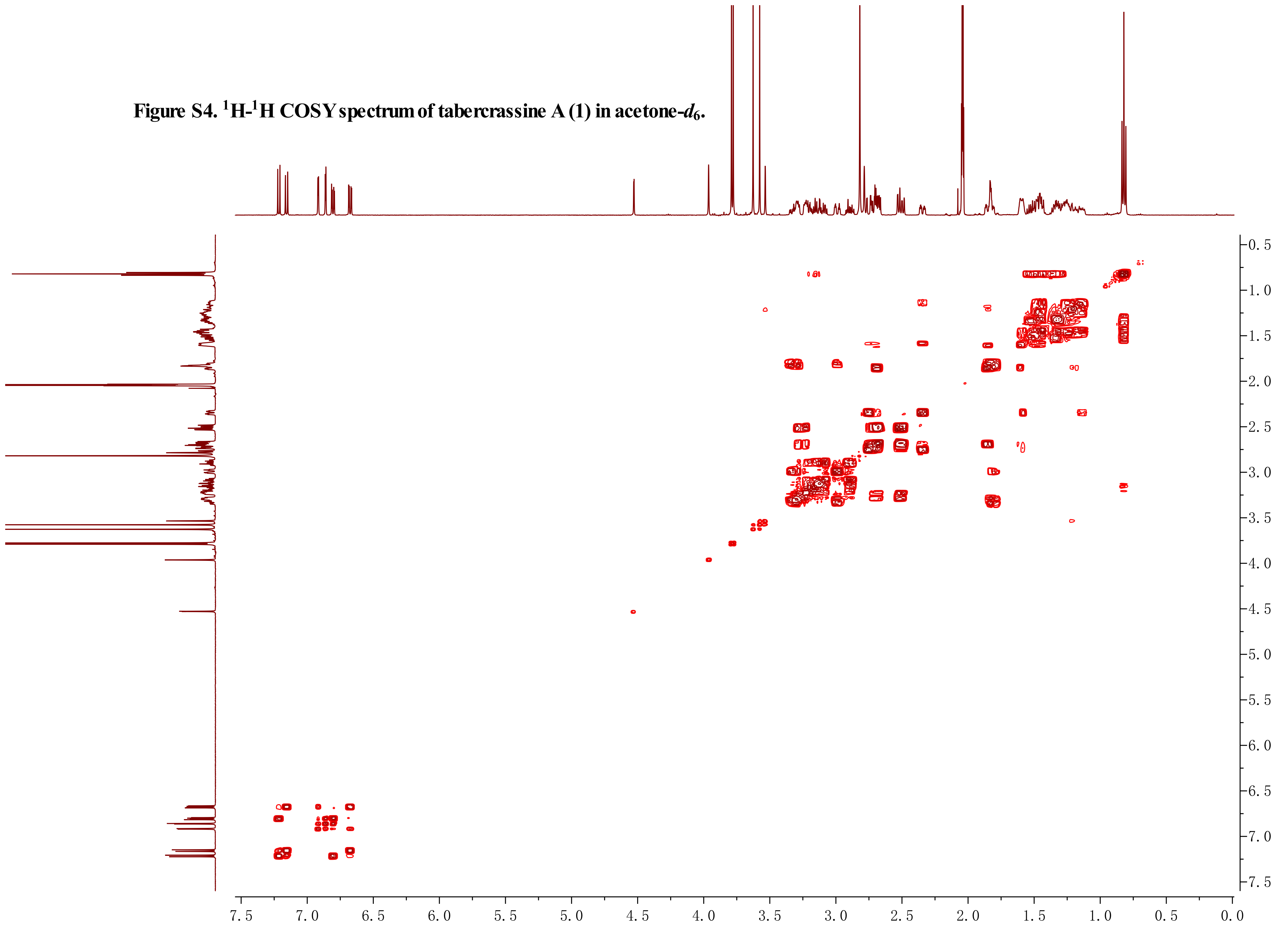

Figure S5. HMBC spectrum of tabercrassine A (1) in acetone-d_6.

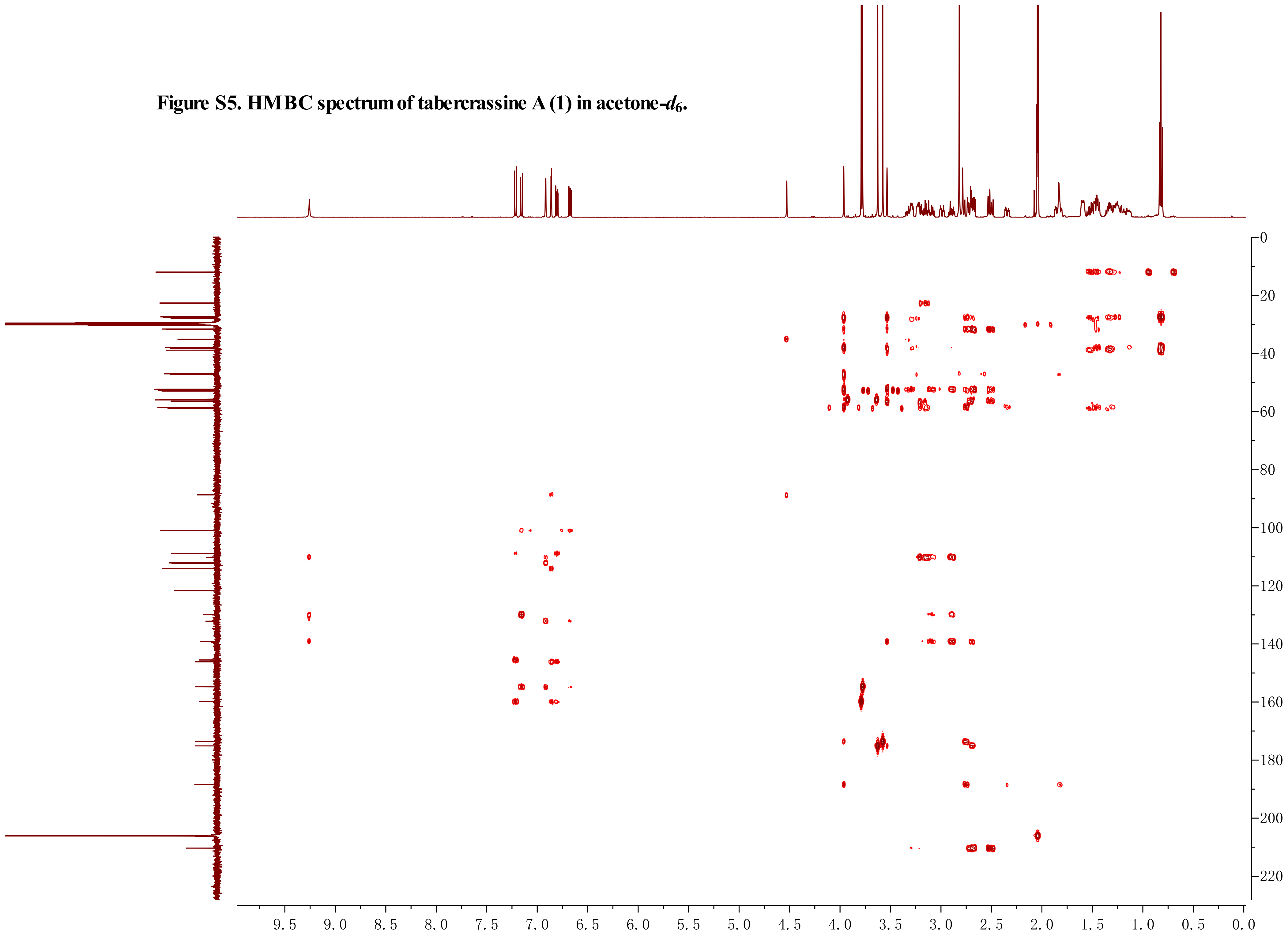

Figure S6. ROESY spectrum of tabercrassine A (1) in acetone-d_6.

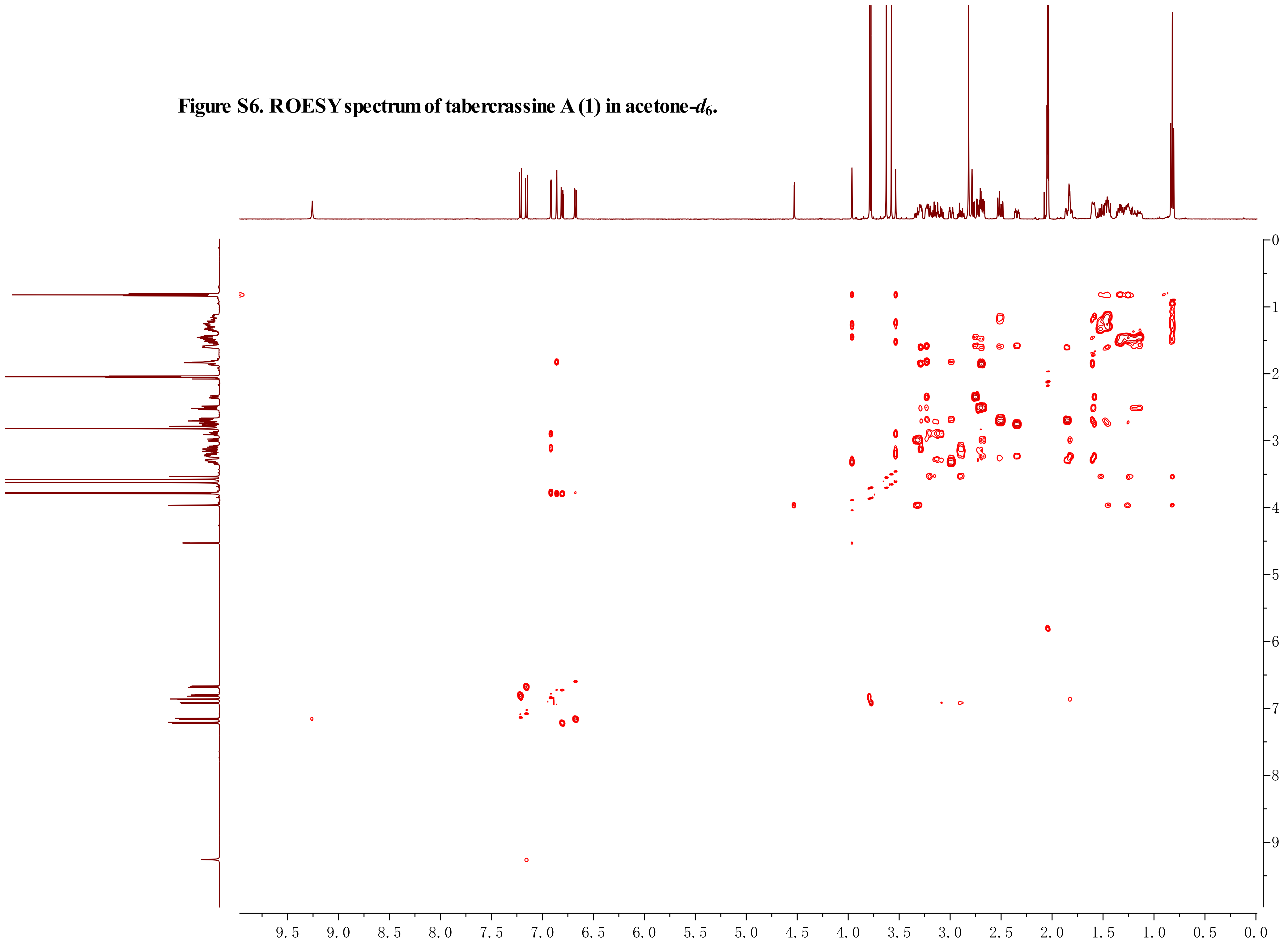

Figure S7. HRESIMS spectrum of tabercrassine A (1).

Qualitative Analysis Report

**Data Filename**	har-30.d	**Sample Name**	har-30
**Sample Type**	Sample	**Position**	P1-A1
**Instrument Name**	Instrument 1	**User Name**	
**Acq Method**	s.m	**Acquired Time**	10/29/2021 11:29:05 AM
**IRM Calibration Status**	Success	**DA Method**	PCDL.m
**Comment**			

Sample Group **Info.**

Acquisition SW 6200 series TOF/6500 series
Version Q-TOF B.05.01 (B5125.2)

User Spectra

Fragmentor Voltage	Collision Energy	Ionization Mode
135	0	ESI

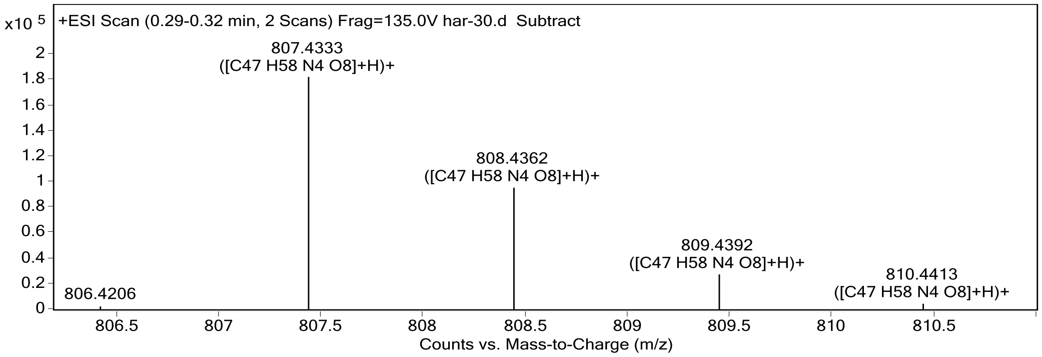

Peak List

m/z	z	Abund	Formula	Ion
367.2016	1	10311.38		
771.3747	1	8700.73		
807.4333	1	182429.08	C47 H58 N4 O8	(M+H)+
808.4362	1	95920.03	C47 H58 N4 O8	(M+H)+
809.4392	1	27782.27	C47 H58 N4 O8	(M+H)+
829.4141	1	28192.25		
830.4182	1	13699.01		
1613.8591	1	33356.17		
1614.8623	1	35436.36		
1615.8654	1	18511.27		

Formula Calculator Element Limits

Element	Min	Max
C	3	60
H	0	200
O	0	10
N	0	5

Formula Calculator Results

Formula	CalculatedMass	CalculatedMz	Mz	Diff. (mDa)	Diff. (ppm)	DBE
C47 H58 N4 O8	806.4255	807.4327	807.4333	-0.60	-0.74	21.0000

--- End Of Report ---

Figure S8. IR spectrum of tabercrassine A (1).

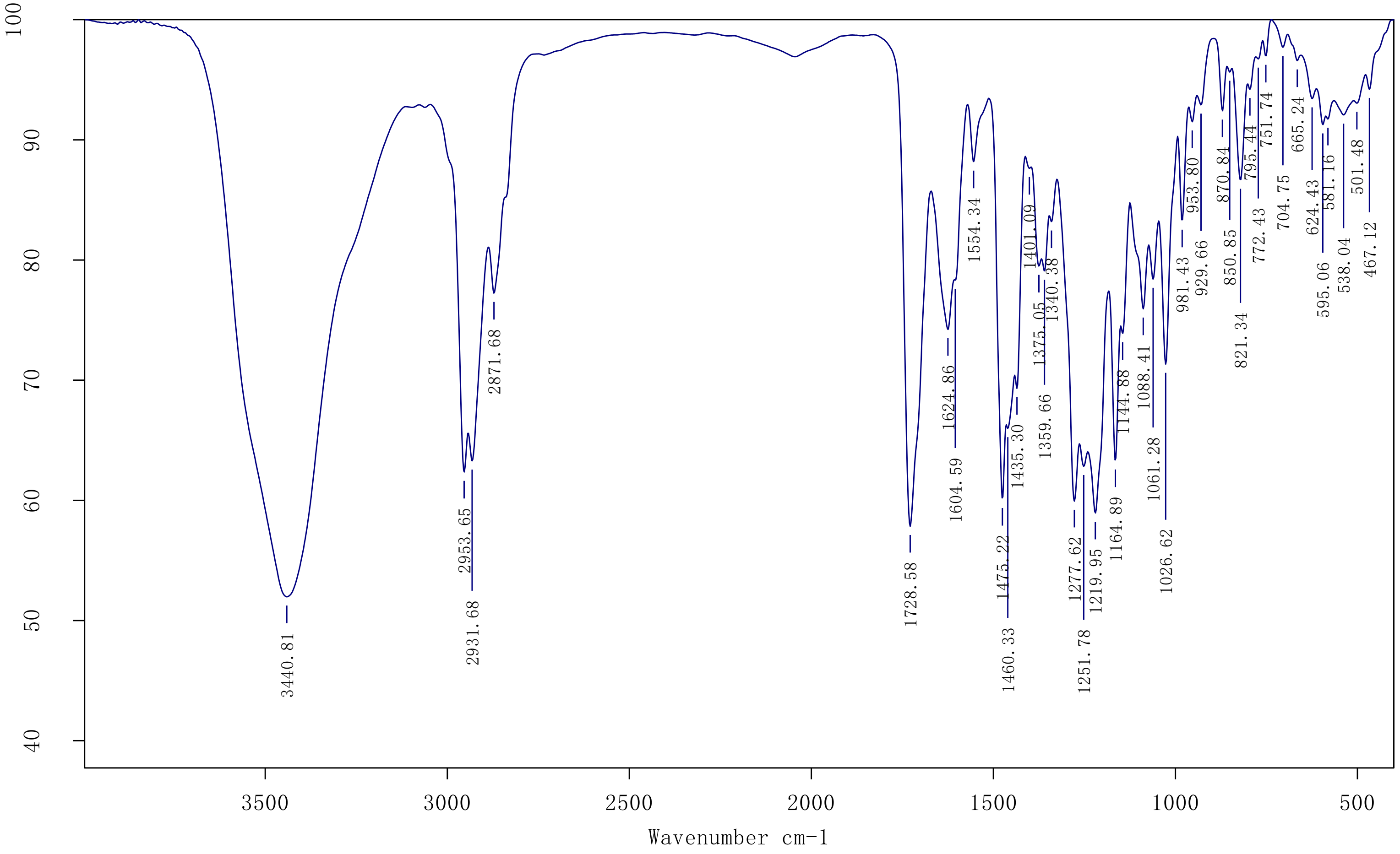

Sample Name: har-30	Resolution: 4	Beamsplitter Setting: KBr
Sample Form: KBr	Aperture Setting: 6 mm	Source Setting: MIR
Path of File: E:\data	Number of Background Scans: 16	Instrument Type: BRUKER VERTEX 70
Date of Measurement: 2022/9/7	Number of Sample Scans: 16	Soft Version: OPUS8.1

Figure S9. ECD spectrum of tabercrassine A (**1**) in MeOH.

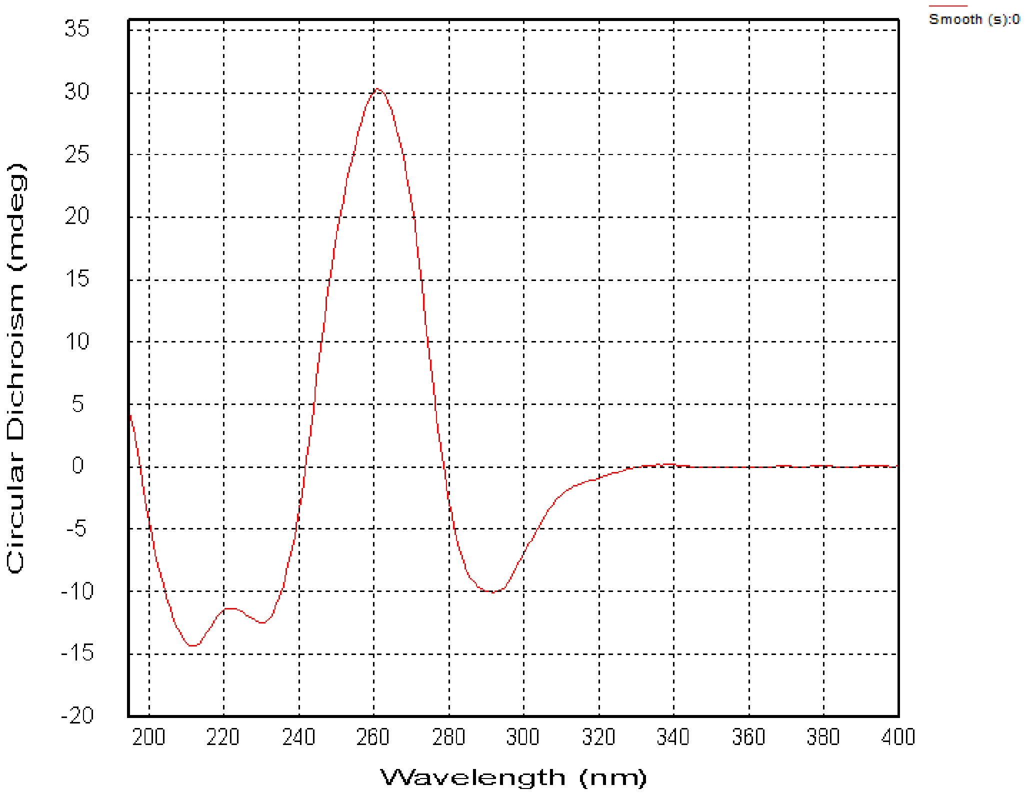

Figure S10.^{1}H NMR spectrum of tabercrassine B (2) in acetone-d_6 (500 MHz).

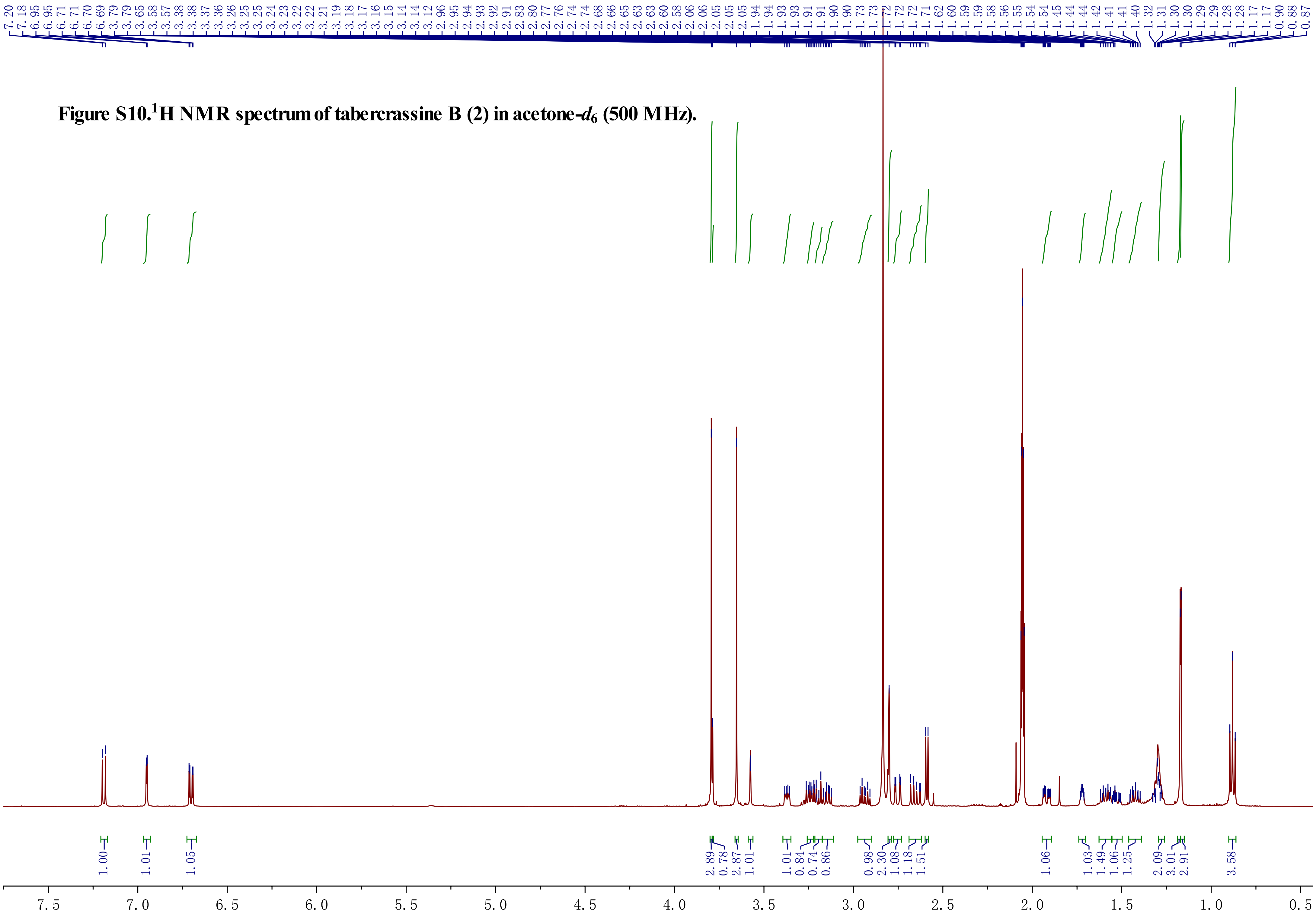

Figure S11. ^{13}**C NMR spectrum of tabercrassine B (2) in acetone-*d*$_6$ (125 MHz).**

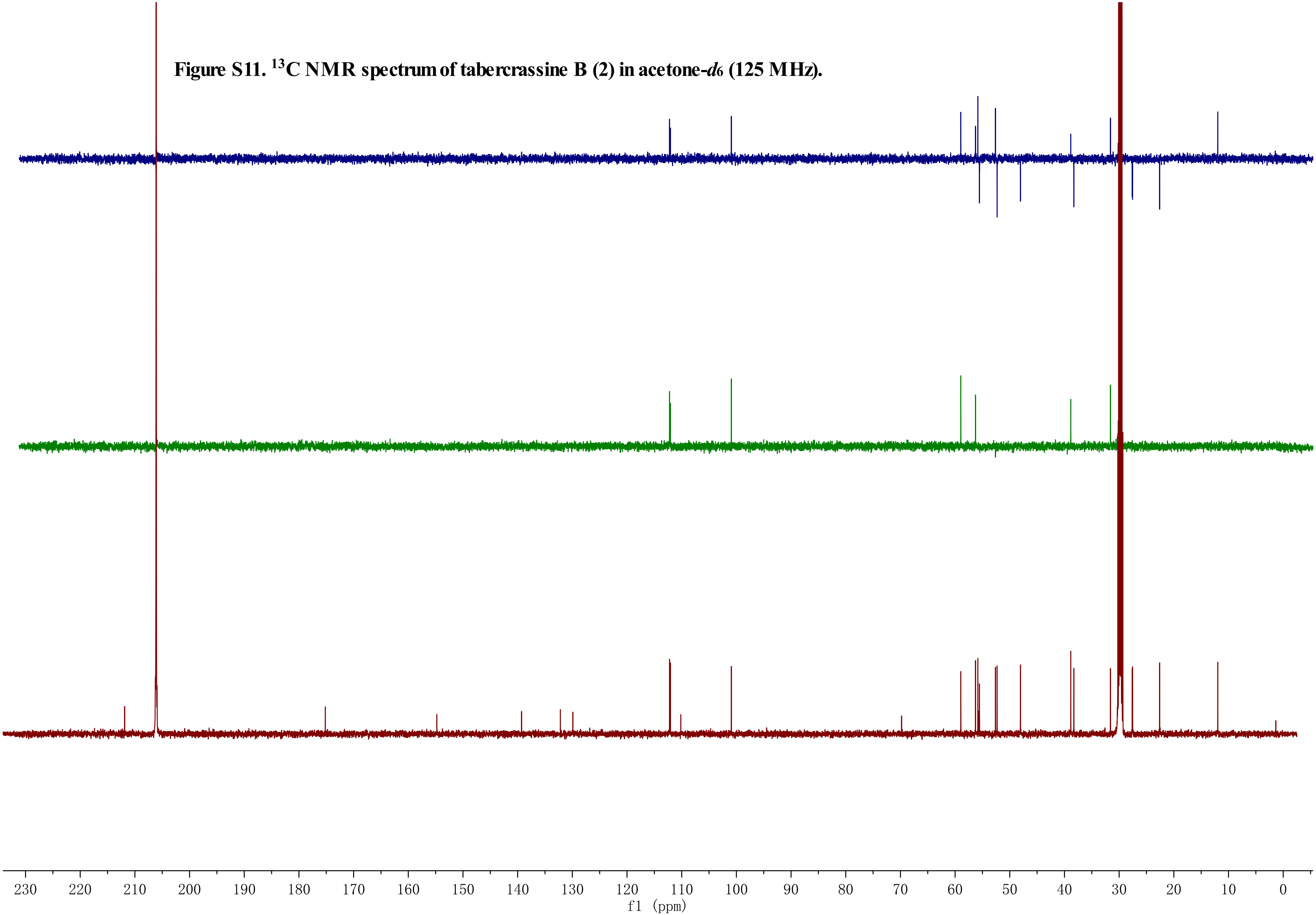

Figure S12. HSQC spectrum of tabercrassine B (2) in acetone-*d*6.

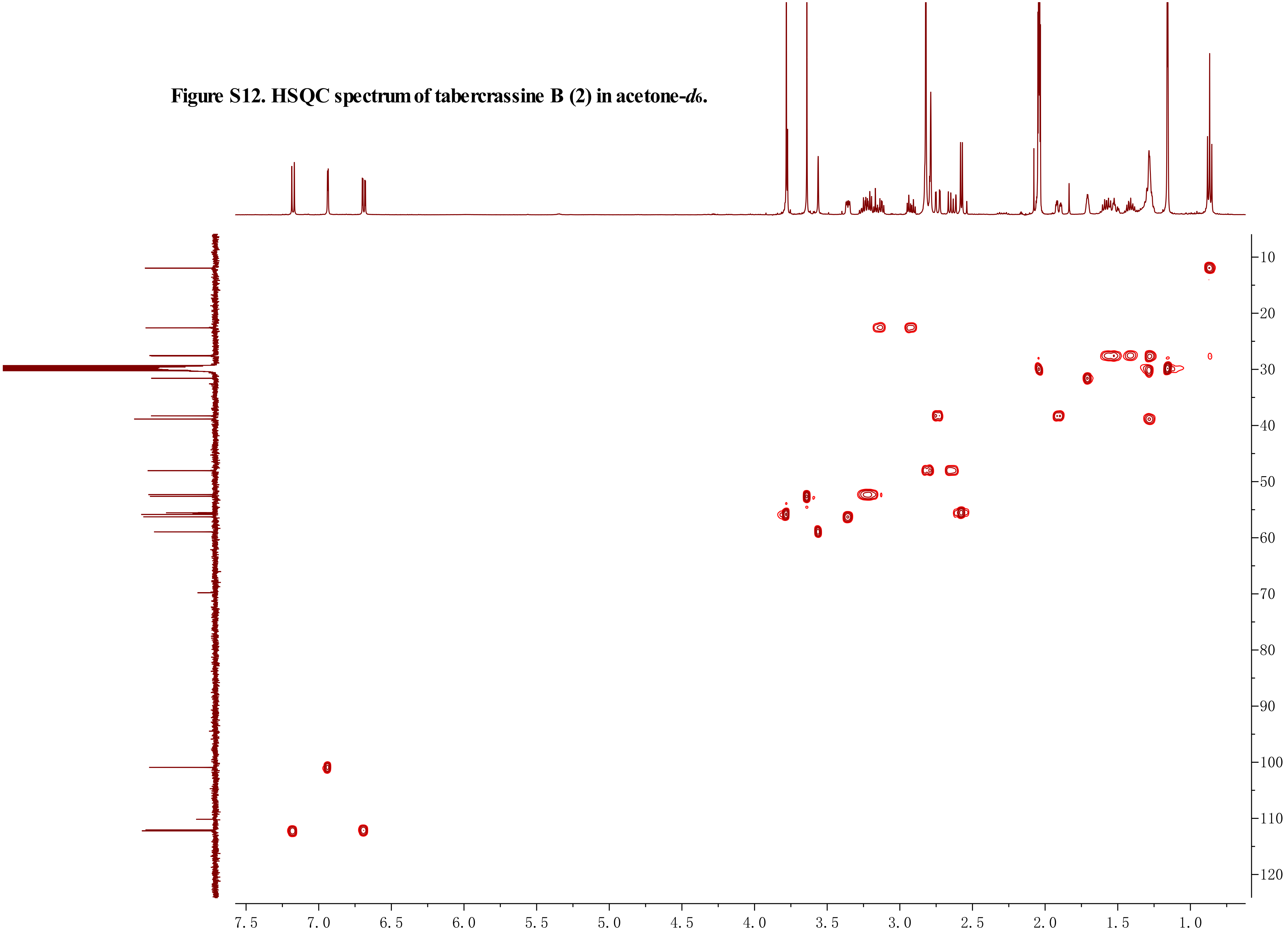

Figure S13. ^{1}H-^{1}H **COSY spectrum of tabercrassine B (2) in acetone-*d*₆.**

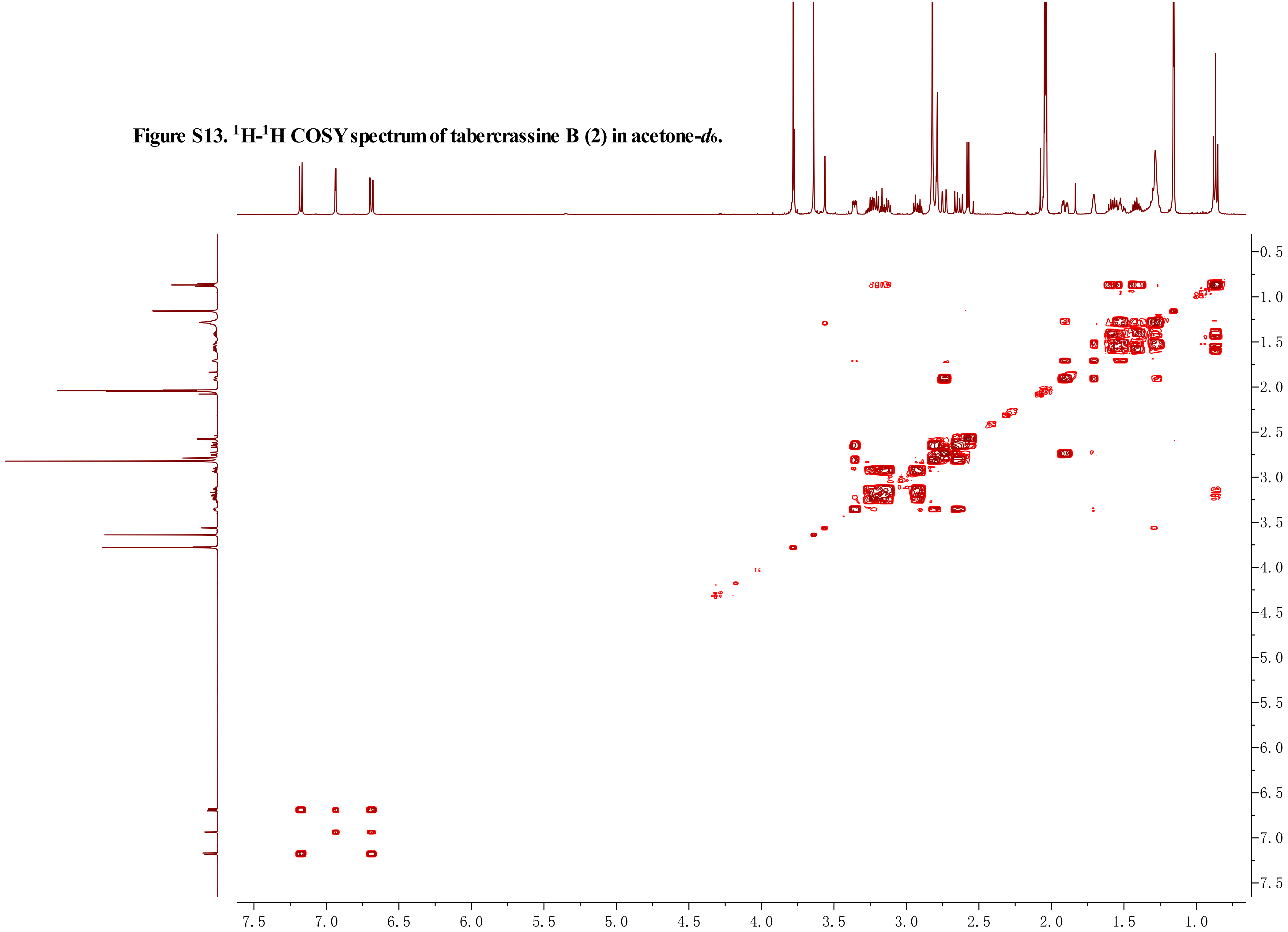

Figure S14. HMBC spectrum of tabercrassine B (2) in acetone-d_6.

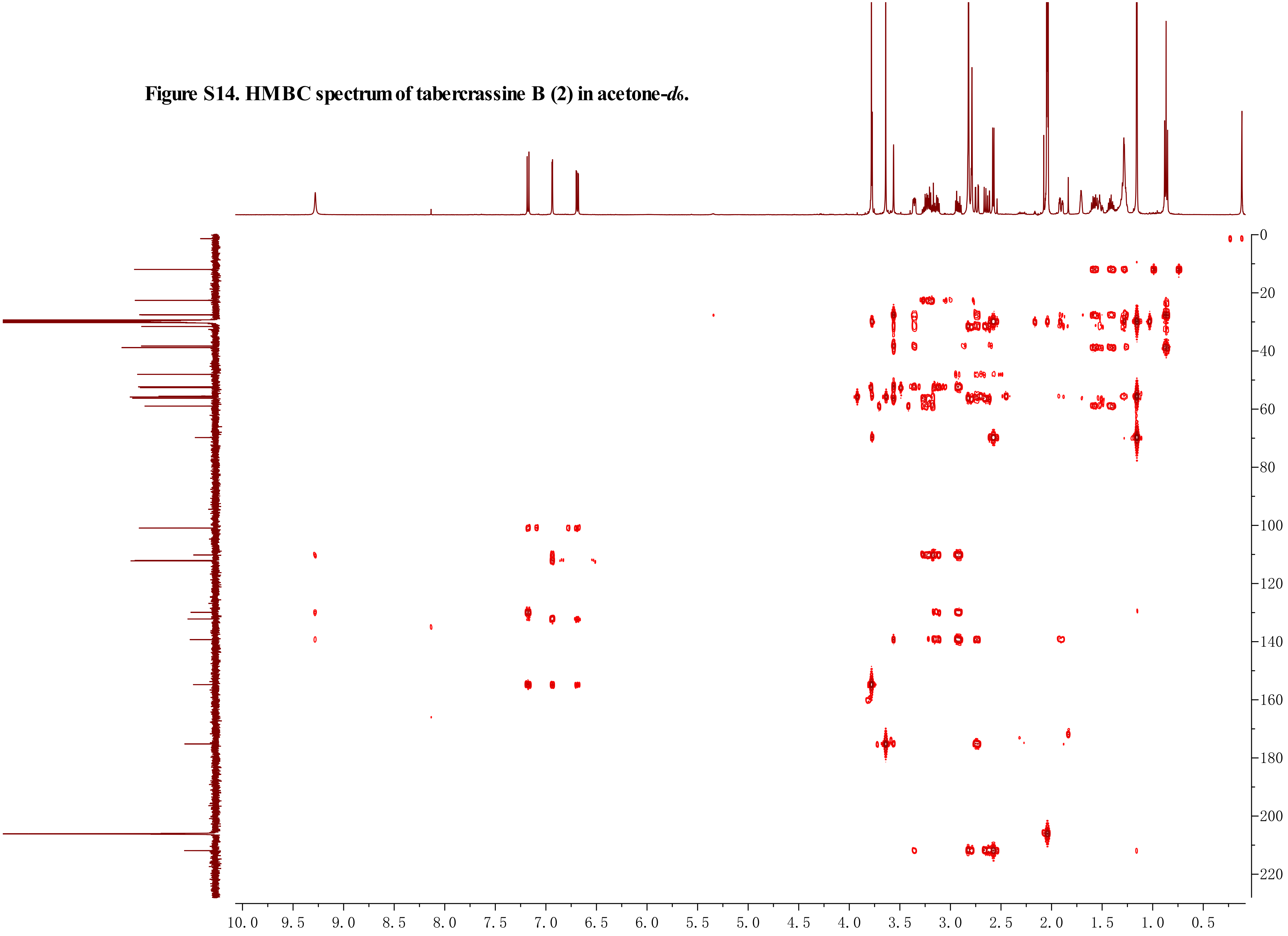

Figure S15. ROESY spectrum of tabercrassine B (2) in acetone-d_6.

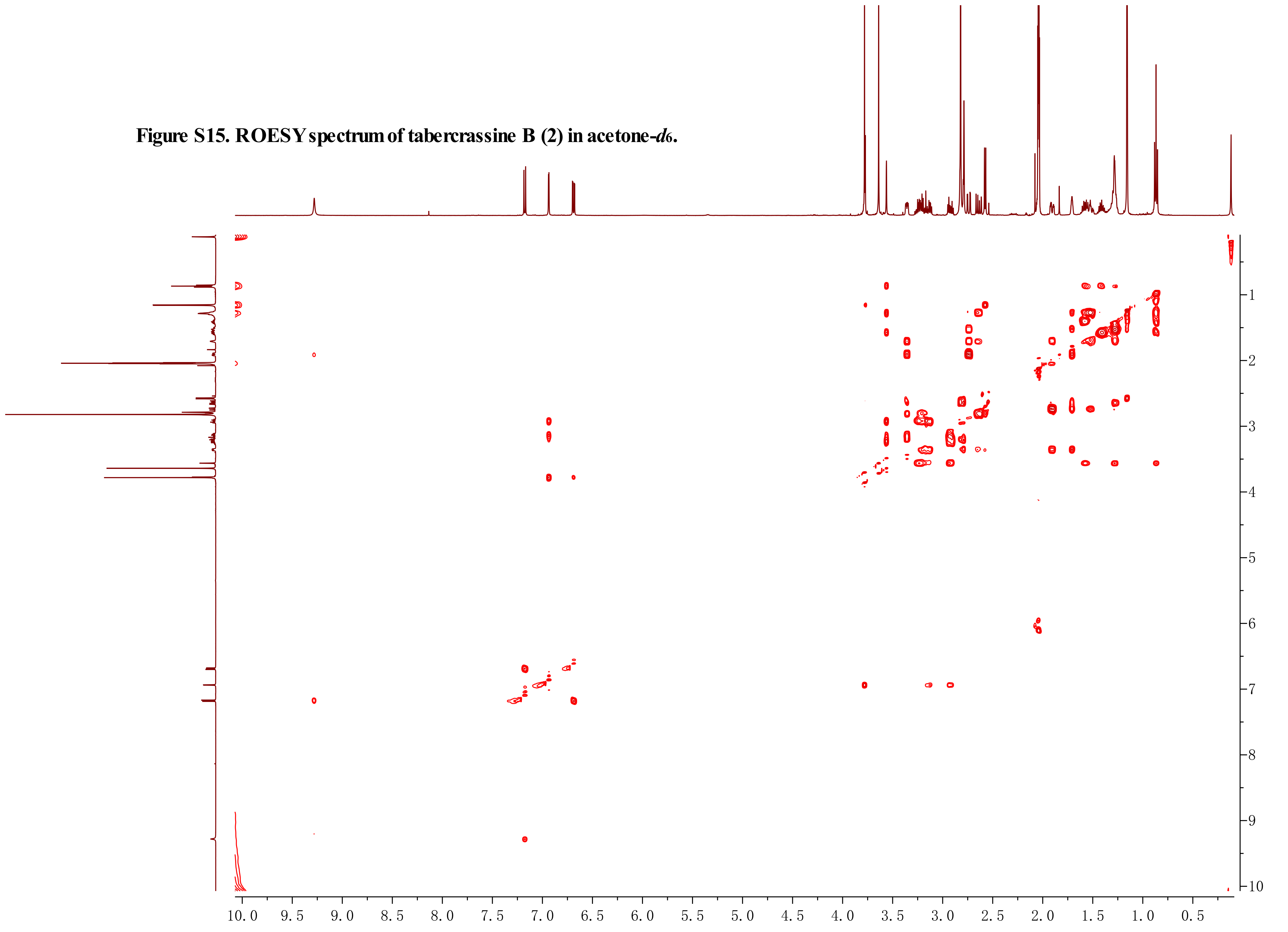

Figure S16. HRESIMS spectrum of tabercrassine B (2).

Qualitative Analysis Report

**Data Filename**	HAR-27.d	**Sample Name**	HAR-27
**Sample Type**	Sample	**Position**	P1-B2
**Instrument Name**	Instrument 1	**User Name**	
**Acq Method**	s.m	**Acquired Time**	11/8/2021 10:58:15 AM
**IRM Calibration Status**	Success	**DA Method**	PCDL.m
**Comment**			

Sample Group **Info.**

Acquisition SW 6200 series TOF/6500 series
Version Q-TOF B.05.01 (B5125.2)

User Spectra

Fragmentor Voltage	Collision Energy	Ionization Mode
135	0	ESI

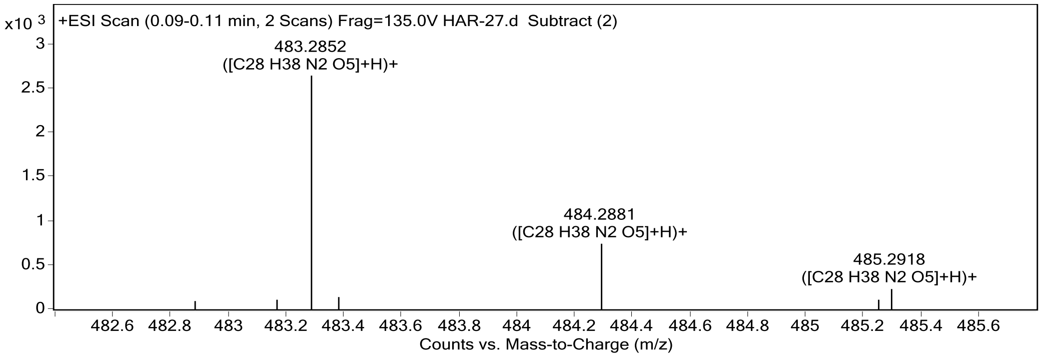

Peak List

m/z	z	Abund	Formula	Ion
81.9374	1	570.76		
95.9531	1	525.11		
120.9682	1	561.94		
150.1117	1	898.52		
153.9787	1	909.54		
155.9739	1	1869.38		
223.1231	1	881.61		
264.9387	1	539.6		
483.2852	1	2651.76	C28 H38 N2 O5	(M+H)+
484.2881	1	750.06	C28 H38 N2 O5	(M+H)+

Formula Calculator Element Limits

Element	Min	Max
C	3	60
H	0	120
O	0	30
N	0	5

Formula Calculator Results

Formula	CalculatedMass	CalculatedMz	Mz	Diff. (mDa)	Diff. (ppm)	DBE
C28 H38 N2 O5	482.2781	483.2853	483.2852	0.10	0.21	11.0000

--- End Of Report ---

Figure S17. IR spectrum of tabercrassine B (2).

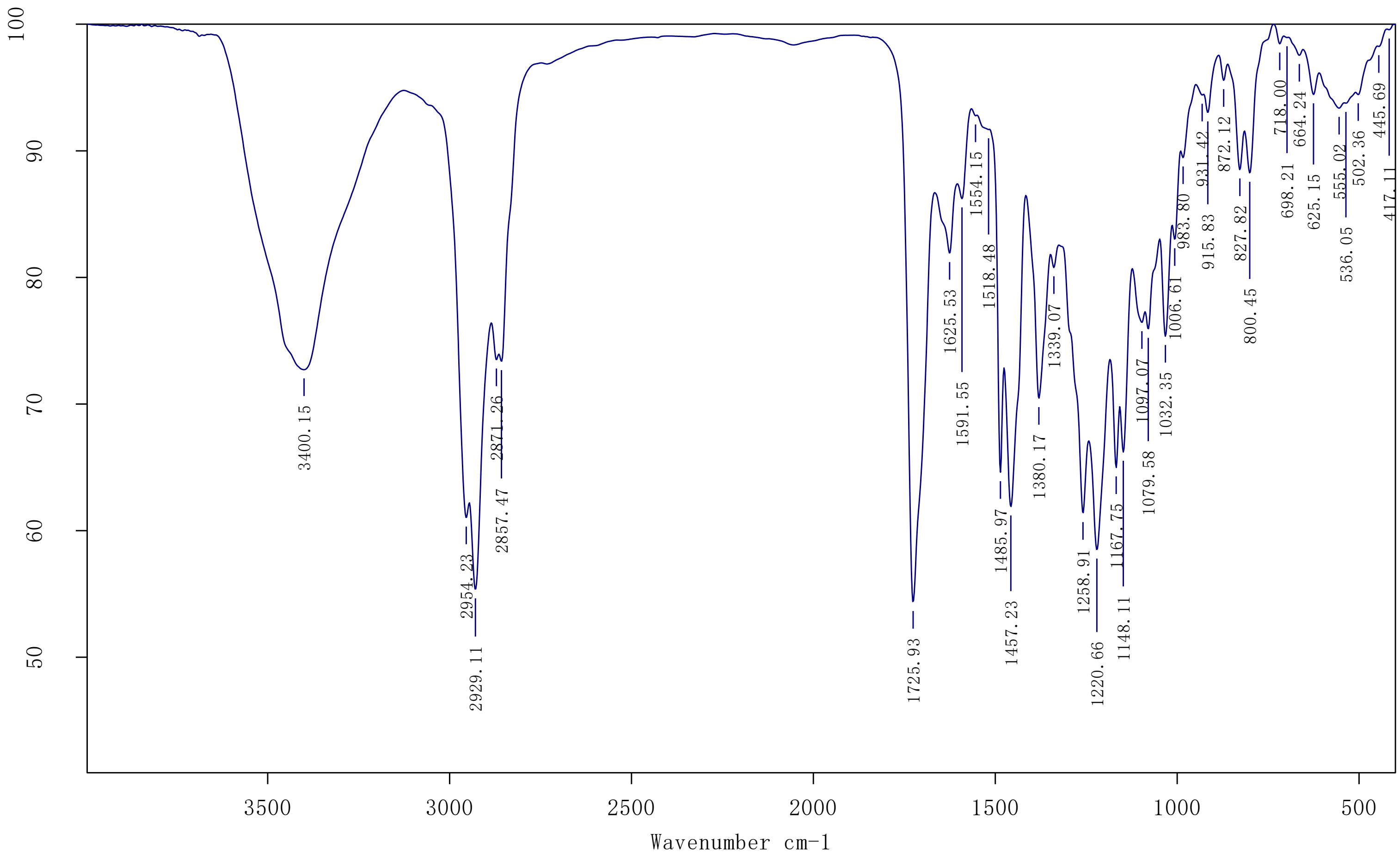

Sample Name: har-27
Sample Form: KBr
Path of File: E:\data
Date of Measurement: 2022/9/9

Resolution: 4
Aperture Setting: 6 mm
Number of Background Scans: 16
Number of Sample Scans: 16

Beamsplitter Setting: KBr
Source Setting: MIR
Instrument Type: BRUKER VERTEX 70
Soft Version: OPUS8.1

Figure S18. ECD spectrum of tabercrassine B (2) in MeOH.

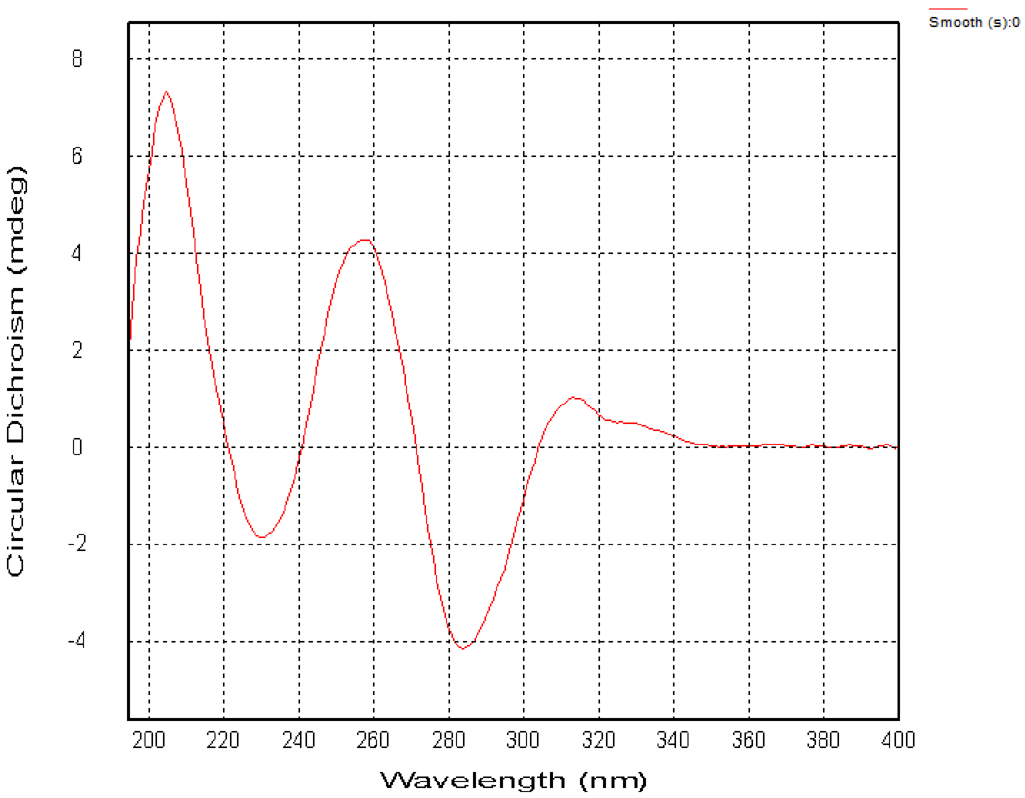

Figure S19. ^{1}**H NMR spectrum of tabercrassine C (3) in acetone-*d*$_6$ (600 MHz).**

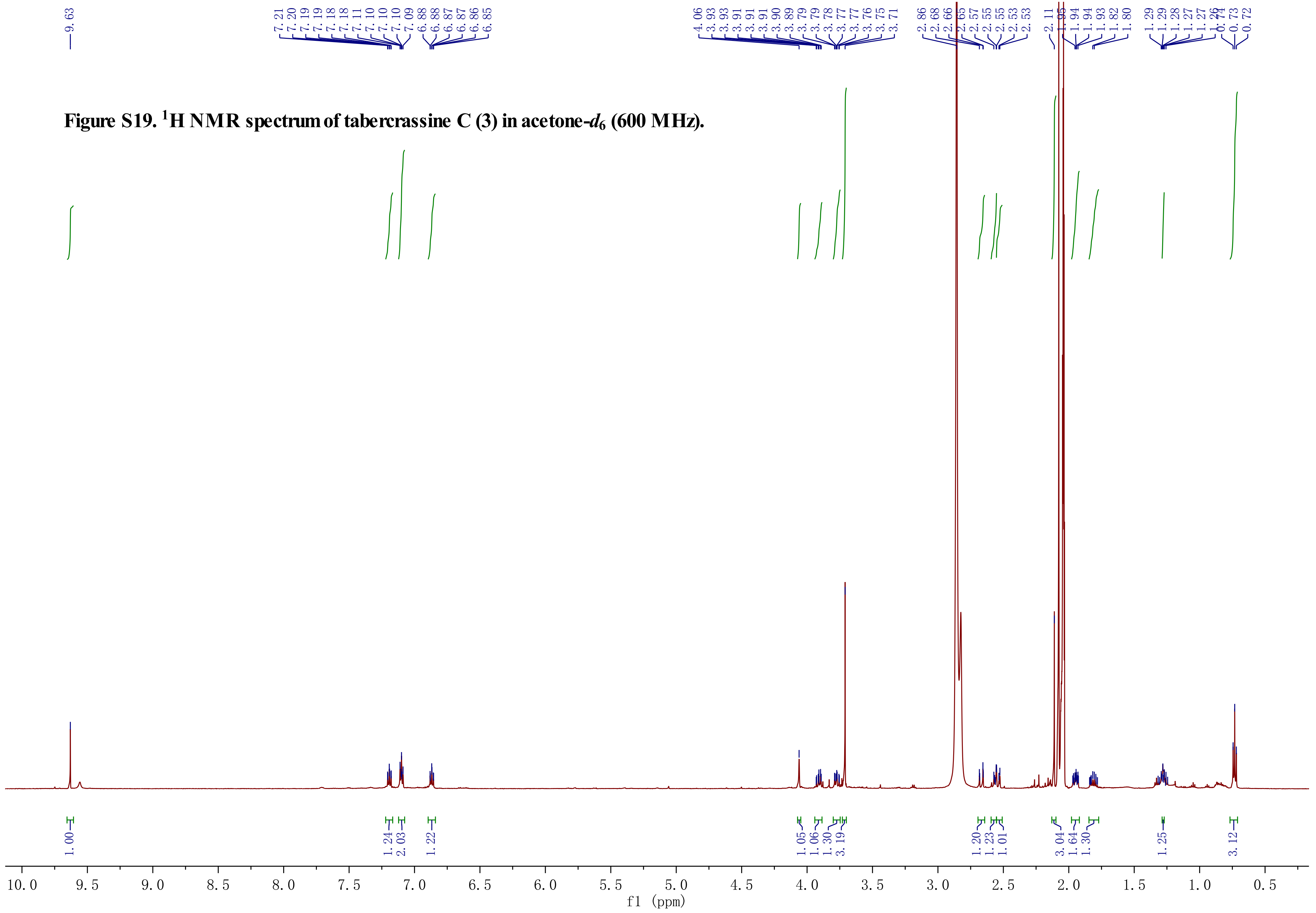

Figure S20. ^{13}C **NMR spectrum of tabercrassine C (3) in acetone-*d*$_6$ (150 MHz).**

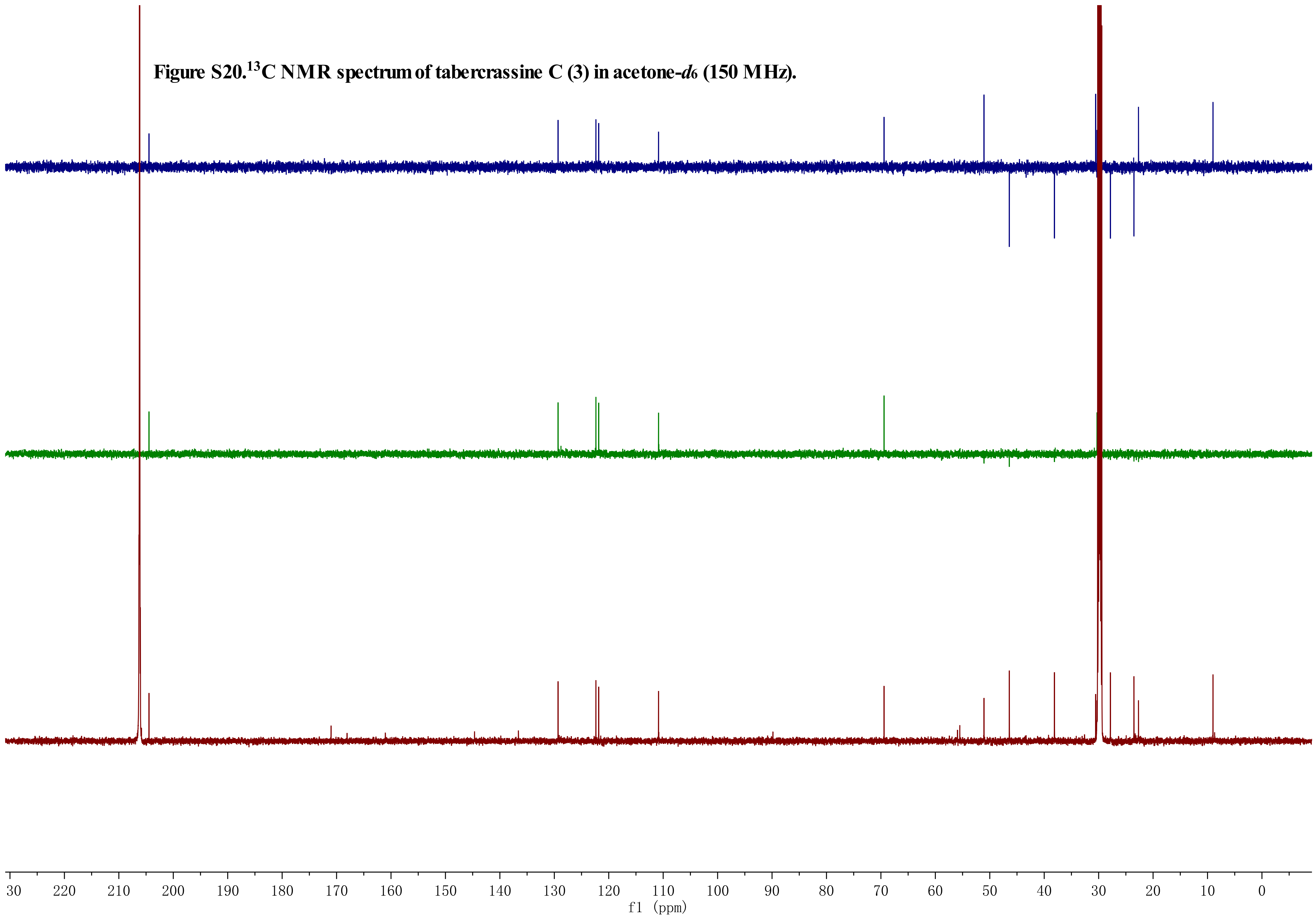

Figure S21. HSQC spectrum of tabercrassine C (3) in acetone-d_6.

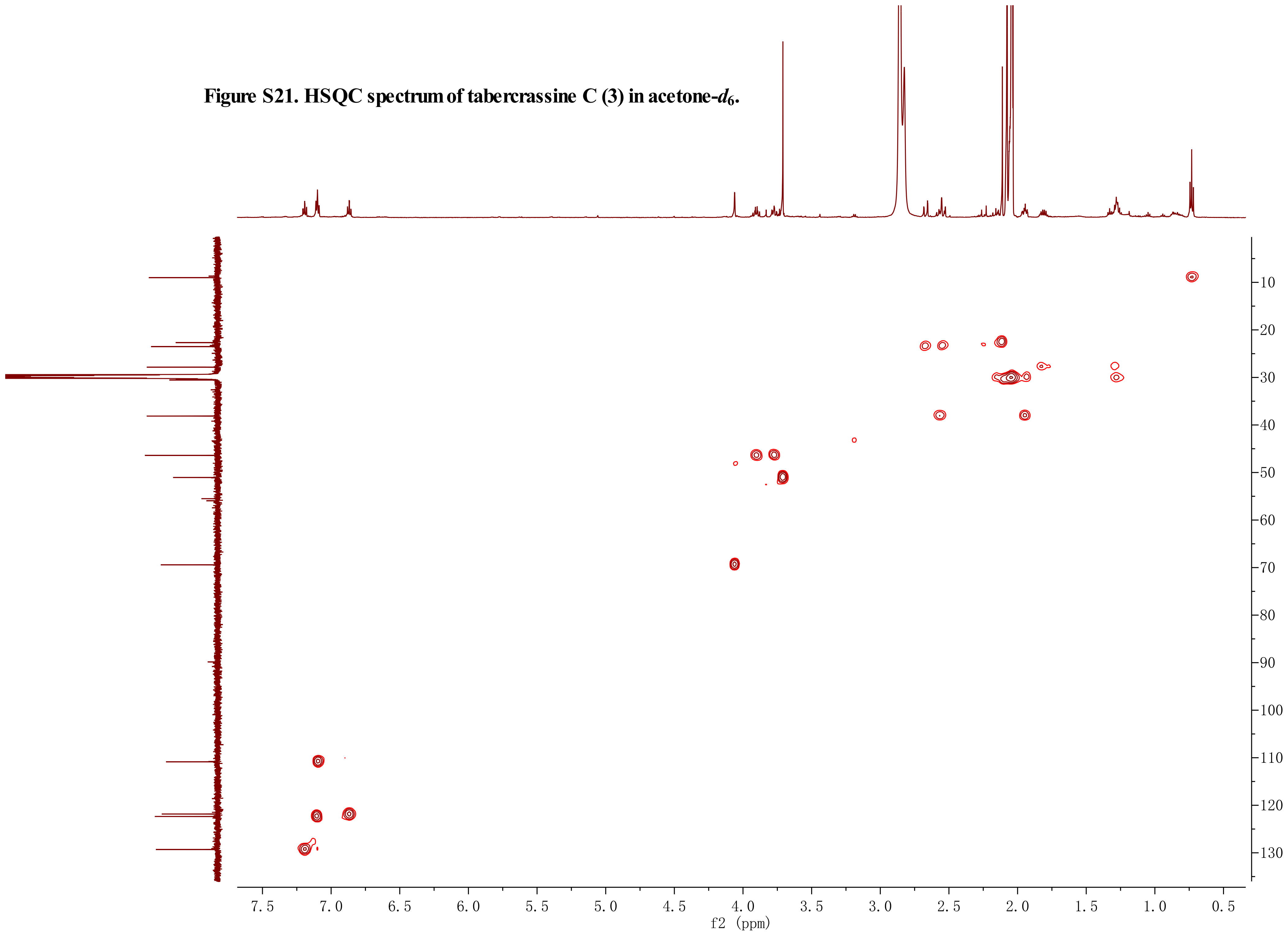

Figure S22. ^{1}H-^{1}H COSY spectrum of tabercrassine C (3) in acetone-d_6.

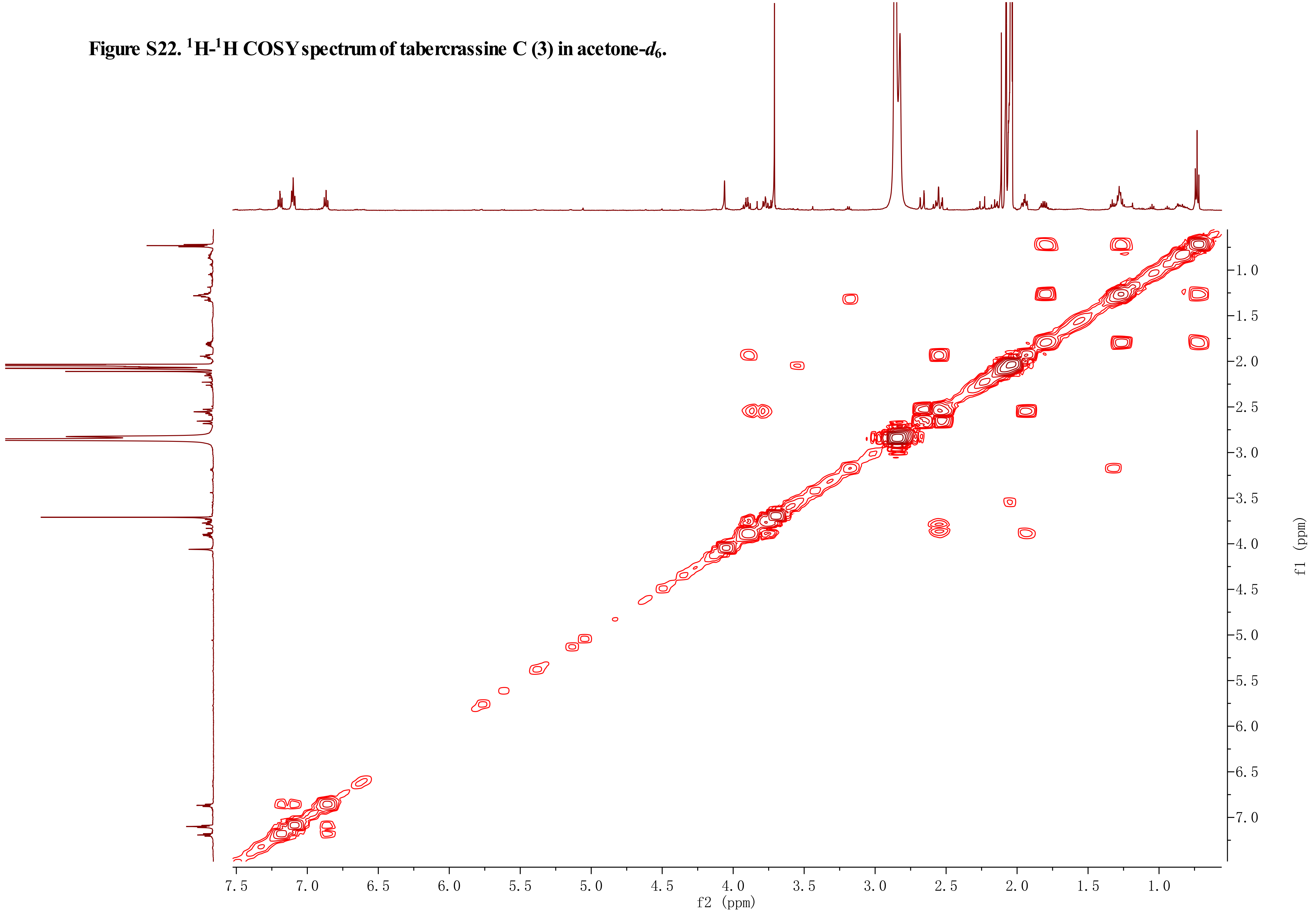

Figure S23. HMBC spectrum of tabercrassine C (3) in acetone-d_6.

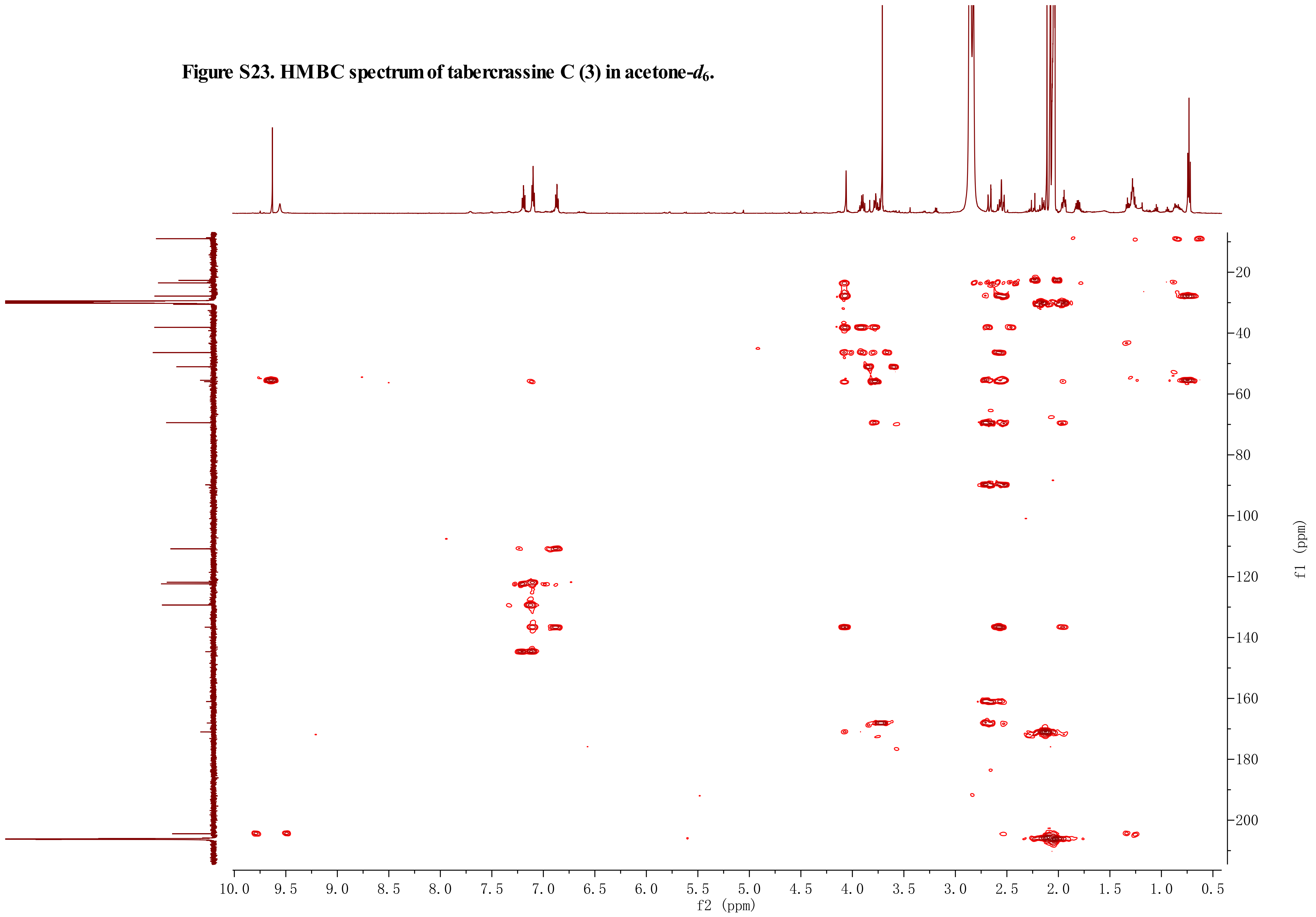

Figure S24. ROESY spectrum of tabercrassine C (3) in acetone-d_6.

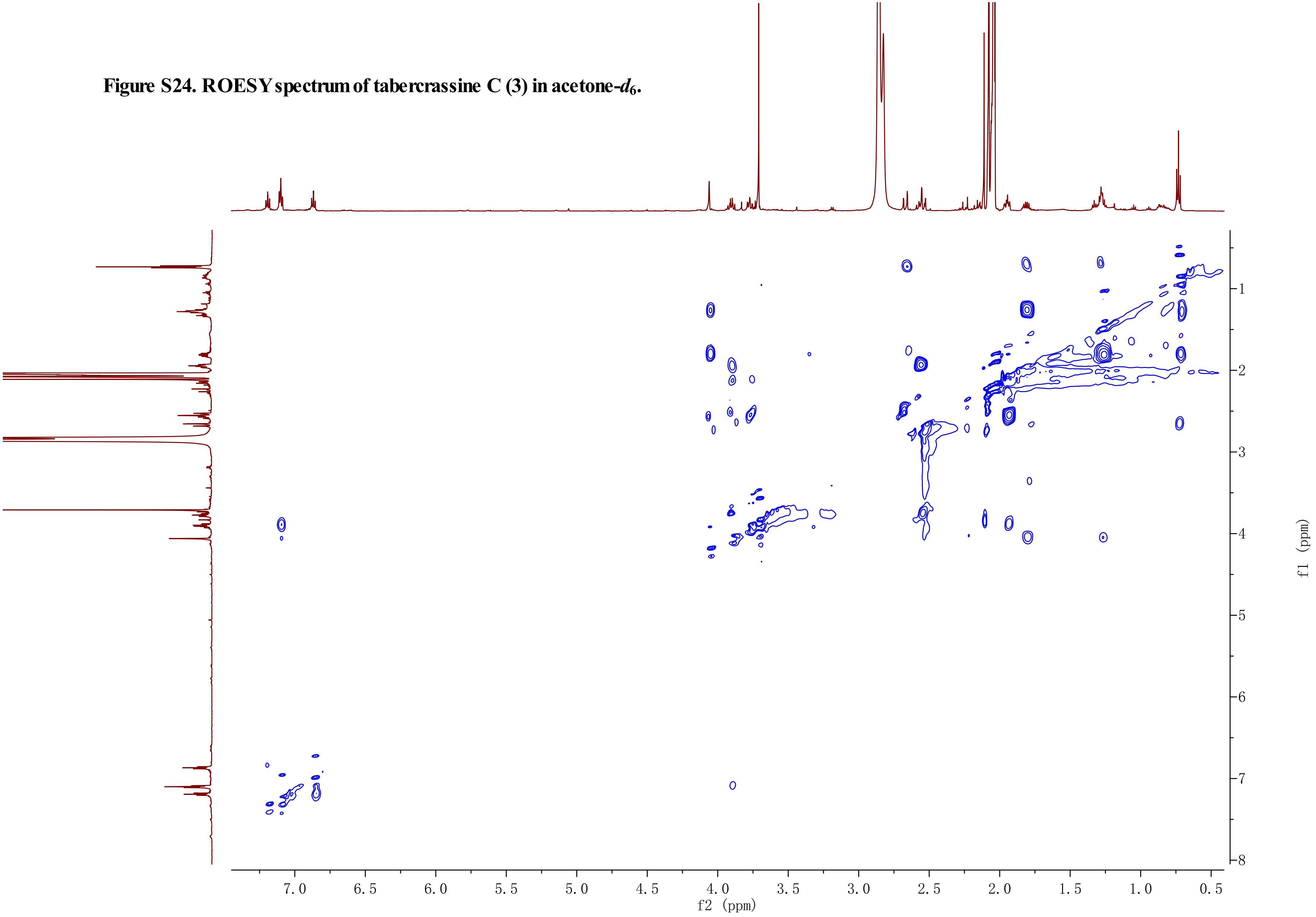

Figure S25. HRESIMS spectrum of tabercrassine C (3)

Qualitative Analysis Report

**Data Filename**	hvf-41.d	**Sample Name**	hvf-41
**Sample Type**	Sample	**Position**	P1-A7
**Instrument Name**	Instrument 1	**User Name**	
**Acq Method**	s.m	**Acquired Time**	8/13/2021 4:42:22 PM
**IRM Calibration Status**	Success	**DA Method**	PCDL.m
**Comment**			

**Sample Group**		**Info.**
**Acquisition SW Version**	6200 series TOF/6500 series Q-TOF B.05.01 (B5125.2)	

User Spectra

Fragmentor Voltage	Collision Energy	Ionization Mode
135	0	ESI

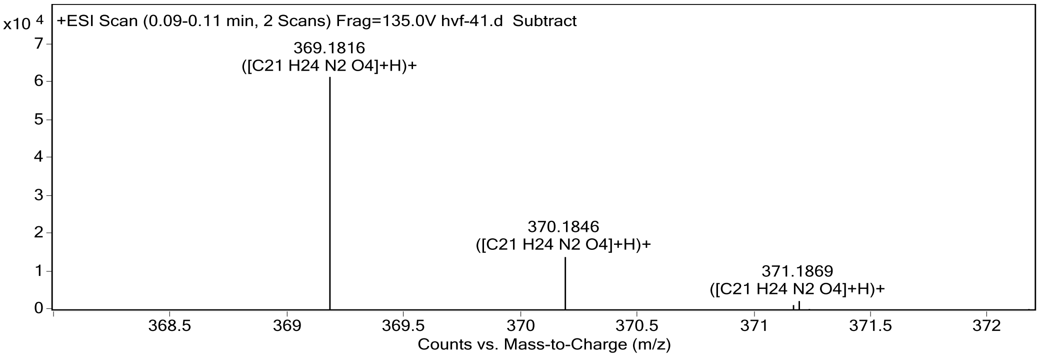

Peak List

m/z	z	Abund	Formula	Ion
274.2747	1	9569.97		
318.3011	1	6406.61		
369.1816	1	61505.75	C21 H24 N2 O4	(M+H)+
370.1846	1	14211.29	C21 H24 N2 O4	(M+H)+
385.1766	1	10916.56		
399.1555	1	8922.11		
401.1714	1	17037.61		
405.1668	1	6267.22		
417.1665	1	13309.22		
855.3073	1	7793.51		

Formula Calculator Element Limits

Element	Min	Max
C	3	60
H	0	120
O	0	30
N	0	3

Formula Calculator Results

Formula	CalculatedMass	CalculatedMz	Mz	Diff. (mDa)	Diff. (ppm)	DBE
C21 H24 N2 O4	368.1736	369.1809	369.1816	-0.70	-1.90	11.0000

--- End Of Report ---

Figure S26. IR spectrum of tabercrassine C (3).

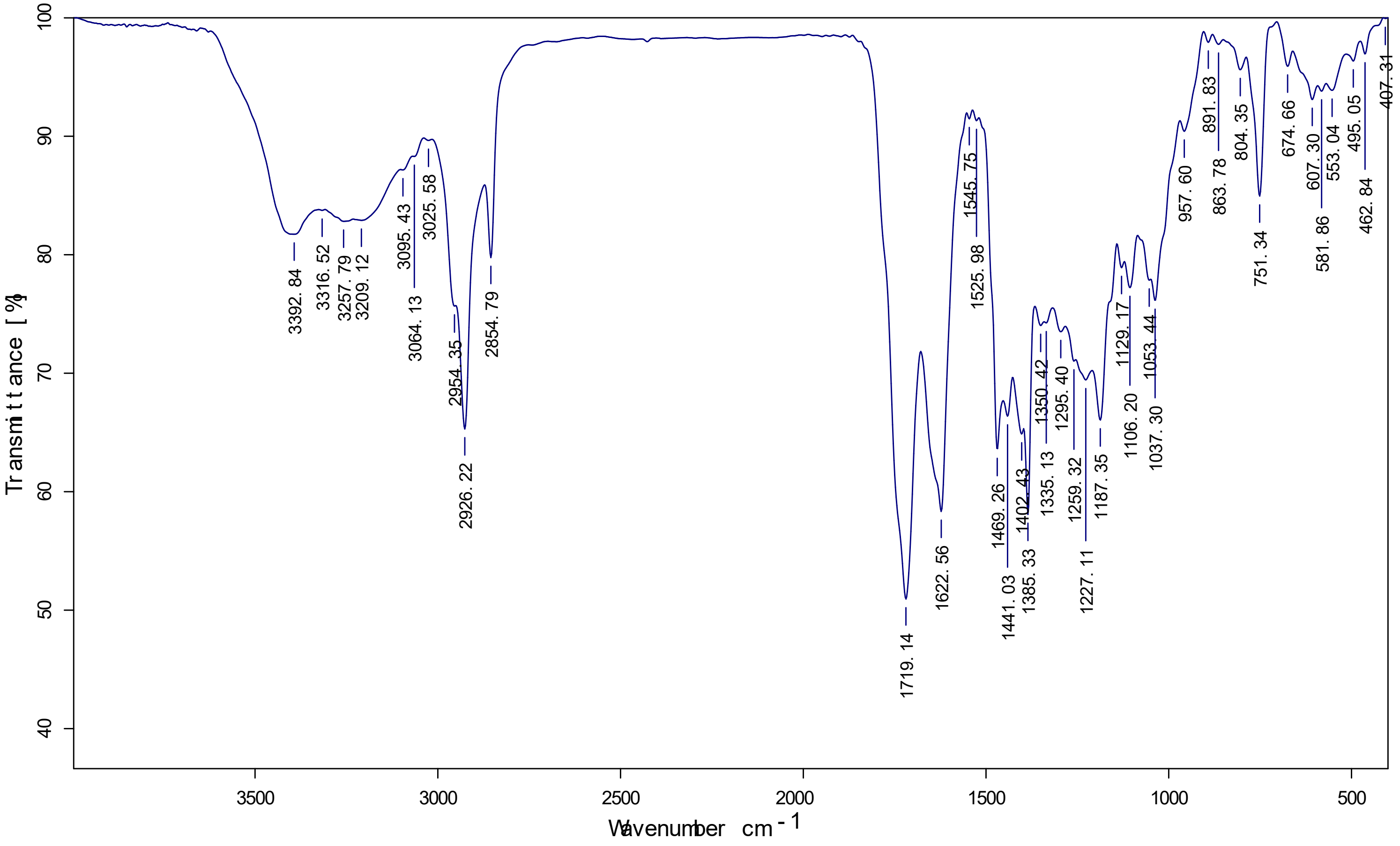

Sample Name: hvf41
Sample Form: KBr
Path of File: E:\data
Date of Measurement: 2022/3/25

Resolution: 4
Aperture Setting: 6 mm
Number of Background Scans: 16
Number of Sample Scans: 16

Beamsplitter Setting: KBr
Source Setting: MIR
Instrument Type: BRUKER VERTEX 70
Soft Version: OPUS8.1

Figure S27. ECD spectrum of tabercrassine C (3) in MeOH.

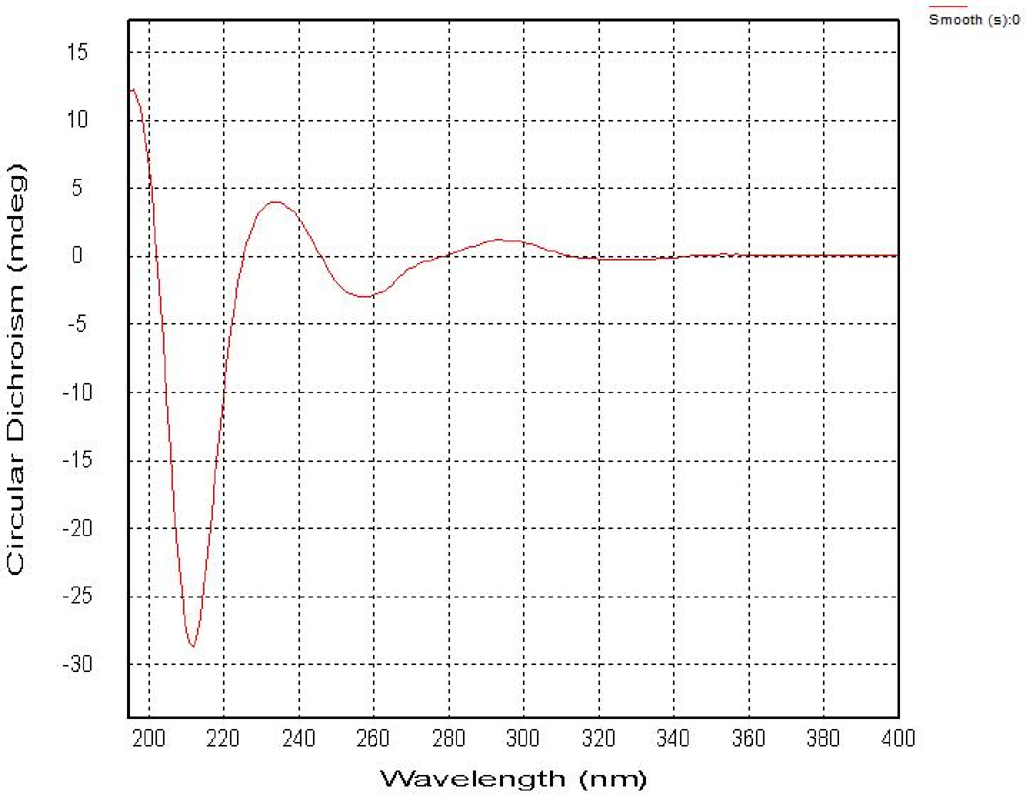

Figure S28. Calculated and experimental ECD of 2 and 3.

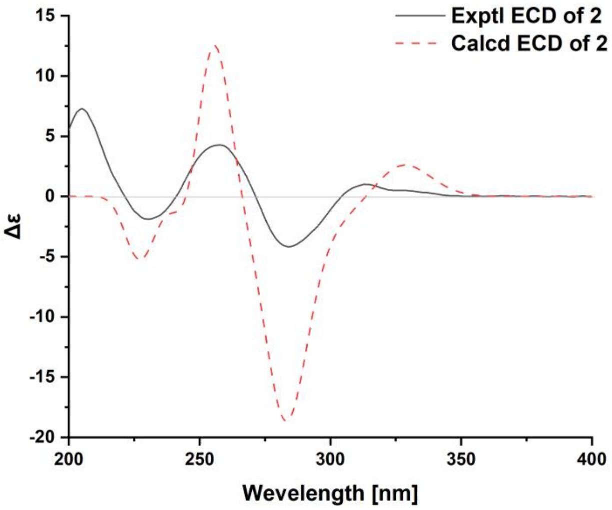

Comparison of the experimental and calculated ECD spectra of **2**.

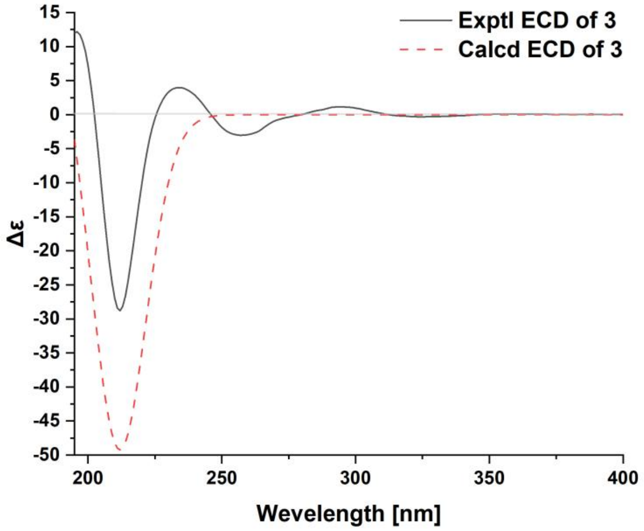

Comparison of the experimental and calculated ECD spectra of **3**.

ECD calculation methods for **2** and **3**:

The CONFLEX[1, 2] searches based on molecular mechanics with MMFF94S force fields were performed for **2** and **3** which gave 41 and 11 stable conformers, respectively. Selected conformers (9 and 5) with distributions higher than 1% were further optimized

by the density functional theory method at the B3LYP/6-31G* level in Gaussian 09 program package,[3] leading to 2 and 4 geometries (ΔE>2 kcal/mol), respectively, which was further checked by frequency calculation and resulted in no imaginary frequencies. The ECD was calculated using TD-DFT-B3LYP/6-31G (d, p) of theory for compounds **2** and **3** on B3LYP/6-31G(d) optimized geometry through the IEFPCM model (in MeOH). The calculated ECD curves were generated using SpecDis 1.60[4].

Standard orientation of **2a** at B3LYP/6-31G(d) level in gas phase:

Center Number	Atomic Number	Atomic Type	Coordinates (Angstroms) X	Y	Z
1	6	0	-4.853816	-2.963797	0.064630
2	6	0	-5.062657	-2.678487	-1.306252
3	6	0	-4.352946	-1.675547	-1.945589
4	6	0	-3.422471	-0.949741	-1.192950
5	6	0	-3.201274	-1.216578	0.181743
6	6	0	-3.930679	-2.243989	0.815614
7	7	0	-2.579670	0.082382	-1.549259
8	6	0	-1.846880	0.487457	-0.443917
9	6	0	-2.199785	-0.289107	0.643206
10	6	0	-0.865253	1.634210	-0.591863
11	6	0	-0.126530	1.927180	0.760391
12	7	0	0.585889	0.738081	1.238007
13	6	0	-0.122278	-0.189993	2.128024
14	6	0	-1.661832	-0.175831	2.041923
15	6	0	1.433371	0.117220	0.203013

16	6	0	0.251166	1.307506	-1.649706
17	6	0	1.604945	1.150391	-0.933925
18	6	0	0.898088	3.068522	0.549375
19	6	0	2.033251	2.513250	-0.358644
20	1	0	-0.848679	2.231281	1.518098
21	1	0	2.347091	0.775204	-1.643701
22	6	0	1.405242	3.636695	1.884194
23	6	0	2.335605	4.844736	1.729675
24	6	0	2.758830	-0.365627	0.819418
25	6	0	3.576255	-1.310829	-0.043344
26	6	0	4.780285	-1.967425	0.622013
27	8	0	3.266183	-1.558584	-1.202735
28	6	0	5.892490	-2.450579	-0.343331
29	6	0	6.520381	-1.267756	-1.103371
30	6	0	6.965026	-3.204149	0.447704
31	8	0	5.351984	-3.399151	-1.261912
32	6	0	-1.729395	2.806015	-1.084145
33	8	0	-1.975938	3.031683	-2.253201
34	8	0	-2.263810	3.516528	-0.070693
35	6	0	-3.183056	4.554490	-0.456620
36	8	0	-5.630448	-3.984792	0.555248
37	6	0	-5.478425	-4.336703	1.916773
38	1	0	-5.796200	-3.272228	-1.841793

39	1	0	-4.517942	-1.465198	-2.998835
40	1	0	-3.772086	-2.456878	1.866801
41	1	0	-2.568791	0.566873	-2.435404
42	1	0	0.161964	-0.004556	3.175732
43	1	0	0.219928	-1.204254	1.887412
44	1	0	-2.030175	-1.012523	2.648631
45	1	0	-2.049277	0.727041	2.534151
46	1	0	0.937441	-0.768573	-0.229141
47	1	0	0.004213	0.385405	-2.184758
48	1	0	0.304661	2.105012	-2.396963
49	1	0	0.383177	3.884730	0.024274
50	1	0	2.256894	3.205466	-1.178684
51	1	0	2.963014	2.409409	0.213985
52	1	0	0.539447	3.930748	2.494294
53	1	0	1.913453	2.835683	2.435843
54	1	0	2.661032	5.217881	2.707361
55	1	0	3.236382	4.596227	1.156487
56	1	0	1.831910	5.669827	1.210618
57	1	0	2.563466	-0.878479	1.772306
58	1	0	3.399320	0.484000	1.095160
59	1	0	5.187777	-1.291977	1.385461
60	1	0	4.391069	-2.842338	1.164693
61	1	0	7.292687	-1.641211	-1.783055

62	1	0	5.771128	-0.740883	-1.703309
63	1	0	6.983103	-0.546488	-0.418171
64	1	0	7.729650	-3.583176	-0.237963
65	1	0	6.523789	-4.060644	0.968042
66	1	0	7.449195	-2.554140	1.184950
67	1	0	4.586133	-2.957435	-1.674092
68	1	0	-3.504094	5.019207	0.475641
69	1	0	-2.688691	5.284429	-1.102217
70	1	0	-4.036581	4.126767	-0.988300
71	1	0	-6.174455	-5.158688	2.096350
72	1	0	-4.456054	-4.673962	2.137514
73	1	0	-5.728156	-3.499482	2.583458

Standard orientation of **2b** at B3LYP/6-31G(d) level in gas phase:

Center Number	Atomic Number	Atomic Type	Coordinates (Angstroms) X	Y	Z
1	6	0	-4.927585	-2.745931	0.414160
2	6	0	-5.173979	-2.520277	-0.958105
3	6	0	-4.443594	-1.566356	-1.664240
4	6	0	-3.469306	-0.844024	-0.979523
5	6	0	-3.210750	-1.053958	0.404703
6	6	0	-3.953713	-2.019397	1.097338
7	7	0	-2.597212	0.136690	-1.407063
8	6	0	-1.812331	0.562559	-0.343490
9	6	0	-2.161044	-0.146515	0.789897
10	6	0	-0.787084	1.651581	-0.580502
11	6	0	0.008894	1.980227	0.732191

12	7	0	0.683386	0.784634	1.249429
13	6	0	-0.036555	-0.060825	2.209449
14	6	0	-1.577258	0.011916	2.163299
15	6	0	1.463754	0.069879	0.221804
16	6	0	0.276195	1.216484	-1.654057
17	6	0	1.644129	1.033944	-0.973683
18	6	0	1.076195	3.059640	0.425980
19	6	0	2.153361	2.402813	-0.485182
20	1	0	-0.675913	2.354213	1.495465
21	1	0	2.343806	0.585796	-1.685518
22	6	0	1.655192	3.669956	1.711463
23	6	0	2.655296	4.804618	1.466116
24	6	0	2.785197	-0.439527	0.825267
25	6	0	3.551968	-1.442667	-0.017486
26	6	0	4.754752	-2.106176	0.644869
27	8	0	3.216101	-1.705598	-1.169857
28	6	0	5.830062	-2.668133	-0.322438
29	6	0	6.383109	-1.556761	-1.234536
30	6	0	6.963511	-3.309455	0.481911
31	8	0	5.269666	-3.724611	-1.105625
32	6	0	-1.612114	2.834185	-1.111212
33	8	0	-1.919669	2.992549	-2.279689
34	8	0	-2.076411	3.629708	-0.122346
35	6	0	-2.974491	4.671794	-0.551521
36	8	0	-5.608218	-3.672420	1.172642
37	6	0	-6.600121	-4.457281	0.534728
38	1	0	-5.934851	-3.089475	-1.479716
39	1	0	-4.635325	-1.399816	-2.721567

40	1	0	-3.797977	-2.219941	2.153874
41	1	0	-2.595116	0.582568	-2.314602
42	1	0	0.284214	0.174278	3.237628
43	1	0	0.262359	-1.100553	2.019401
44	1	0	-1.962866	-0.776571	2.823151
45	1	0	-1.910389	0.956228	2.619799
46	1	0	0.914064	-0.815611	-0.145260
47	1	0	-0.032377	0.278411	-2.128344
48	1	0	0.341326	1.969602	-2.447121
49	1	0	0.577962	3.869582	-0.127192
50	1	0	2.385968	3.040374	-1.347356
51	1	0	3.093864	2.281973	0.068478
52	1	0	0.826217	4.051461	2.326639
53	1	0	2.126932	2.867113	2.294745
54	1	0	3.026867	5.213349	2.413818
55	1	0	3.525850	4.465206	0.890815
56	1	0	2.192506	5.629436	0.907482
57	1	0	2.592293	-0.916101	1.798887
58	1	0	3.461530	0.395640	1.061466
59	1	0	5.198542	-1.399195	1.359347
60	1	0	4.357705	-2.939202	1.247232
61	1	0	7.123126	-1.983195	-1.920546
62	1	0	5.585713	-1.105824	-1.836833
63	1	0	6.867163	-0.761276	-0.652725
64	1	0	7.705878	-3.733269	-0.203702
65	1	0	6.576347	-4.122950	1.106244
66	1	0	7.461923	-2.575114	1.126227
67	1	0	4.464555	-3.340074	-1.510484

68	1	0	-3.244071	5.212178	0.357153
69	1	0	-2.478265	5.334994	-1.266284
70	1	0	-3.861260	4.237444	-1.022732
71	1	0	-6.998515	-5.119299	1.307995
72	1	0	-7.416178	-3.837971	0.133450
73	1	0	-6.178708	-5.064599	-0.280078

Standard orientation of **3a** at B3LYP/6-31G(d) level in gas phase

Center Number	Atomic Number	Atomic Type	Coordinates (Angstroms) X	Y	Z
1	6	0	-2.739662	3.640289	0.771878
2	6	0	-1.715003	4.589028	0.832621
3	6	0	-0.393297	4.253381	0.506773
4	6	0	-2.464373	2.321908	0.370931
5	6	0	-1.160051	1.973807	0.045258
6	6	0	-0.137382	2.940038	0.121532
7	7	0	1.083635	2.350297	-0.232080
8	6	0	0.936676	0.999691	-0.419401
9	6	0	-0.545918	0.675809	-0.466937
10	6	0	1.891103	0.032625	-0.509327
11	6	0	1.406247	-1.397549	-0.580695
12	6	0	0.260673	-1.675903	0.442454
13	6	0	-0.932054	-0.622122	0.306419
14	7	0	-2.125027	-1.077894	-0.417371
15	6	0	-2.293419	-0.474453	-1.743585
16	6	0	-1.019936	0.363049	-1.925485
17	6	0	-3.000225	-1.932248	0.198461
18	8	0	-2.762177	-2.377825	1.323151
19	6	0	-4.248312	-2.313880	-0.582239

20	6	0	0.758405	-1.604955	1.910064
21	6	0	1.989218	-2.459745	2.241235
22	6	0	-0.272793	-3.085943	0.161793
23	8	0	-0.521570	-3.494747	-0.955327
24	6	0	3.297279	0.420988	-0.422505
25	8	0	3.700509	1.580777	-0.316502
26	8	0	4.148504	-0.634658	-0.461109
27	6	0	5.541179	-0.308243	-0.373263
28	1	0	-3.755586	3.920912	1.038917
29	1	0	-1.940478	5.606753	1.144140
30	1	0	0.403409	4.991452	0.563582
31	1	0	-3.263598	1.584278	0.335014
32	1	0	2.012286	2.737621	-0.091312
33	1	0	1.042125	-1.666827	-1.580500
34	1	0	2.238839	-2.076887	-0.383592
35	1	0	-1.242843	-0.377220	1.325695
36	1	0	-2.386328	-1.241896	-2.521520
37	1	0	-3.194662	0.154271	-1.774881
38	1	0	-0.255857	-0.218221	-2.449280
39	1	0	-1.196308	1.279906	-2.495187
40	1	0	-3.979795	-2.896041	-1.473328
41	1	0	-4.879024	-2.925844	0.065609
42	1	0	-4.813213	-1.434830	-0.917251
43	1	0	0.973600	-0.558344	2.163411
44	1	0	-0.083305	-1.908971	2.548574
45	1	0	2.181299	-2.440088	3.320951
46	1	0	1.851014	-3.510580	1.953386
47	1	0	2.890792	-2.091945	1.738067

48	1	0	-0.460935	-3.717207	1.055736
49	1	0	5.840920	0.341864	-1.202271
50	1	0	5.764108	0.201041	0.570608
51	1	0	6.071020	-1.261841	-0.426014

Standard orientation of **3b** at B3LYP/6-31G(d) level in gas phase

Center Number	Atomic Number	Atomic Type	Coordinates (Angstroms) X	Y	Z
1	6	0	-0.932127	4.351186	0.816378
2	6	0	0.397815	4.770339	0.880105
3	6	0	1.447074	3.908471	0.538859
4	6	0	-1.246275	3.049508	0.397814
5	6	0	-0.217532	2.183153	0.057073
6	6	0	1.116279	2.619239	0.136353
7	7	0	1.967097	1.571833	-0.236725
8	6	0	1.261268	0.409748	-0.438396
9	6	0	-0.218825	0.756533	-0.472323
10	6	0	1.691544	-0.876191	-0.547128
11	6	0	0.627461	-1.945862	-0.622657
12	6	0	-0.510208	-1.719531	0.417930
13	6	0	-1.122256	-0.250288	0.303325
14	7	0	-2.400846	-0.131027	-0.401678
15	6	0	-2.304955	0.478238	-1.730913
16	6	0	-0.798765	0.692486	-1.925468
17	6	0	-3.546687	-0.544280	0.217925
18	8	0	-3.500249	-1.080396	1.326567
19	6	0	-4.845610	-0.358898	-0.542436
20	6	0	-0.019237	-1.893520	1.877594
21	6	0	0.728710	-3.198486	2.178697

22	6	0	-1.604719	-2.747629	0.123469
23	8	0	-2.024537	-2.968052	-0.995314
24	6	0	3.087257	-1.296553	-0.460509
25	8	0	3.479182	-2.452269	-0.438306
26	8	0	3.970753	-0.244437	-0.376848
27	6	0	5.351306	-0.627419	-0.273264
28	1	0	-1.729111	5.034140	1.095217
29	1	0	0.628700	5.780897	1.205884
30	1	0	2.481695	4.234468	0.598207
31	1	0	-2.281963	2.723057	0.361169
32	1	0	2.966168	1.542278	-0.083724
33	1	0	0.167436	-2.011490	-1.615442
34	1	0	1.098807	-2.916666	-0.455409
35	1	0	-1.283834	0.093148	1.327446
36	1	0	-2.721775	-0.180749	-2.500282
37	1	0	-2.853124	1.429231	-1.762227
38	1	0	-0.362247	-0.152001	-2.463118
39	1	0	-0.572928	1.602766	-2.486321
40	1	0	-4.859252	-0.998958	-1.432966
41	1	0	-5.668600	-0.646346	0.113411
42	1	0	-4.986333	0.676479	-0.873630
43	1	0	0.625622	-1.045129	2.139707
44	1	0	-0.904458	-1.815352	2.523495
45	1	0	0.925763	-3.279491	3.253728
46	1	0	0.146338	-4.081701	1.887299
47	1	0	1.691274	-3.248882	1.659931
48	1	0	-2.009235	-3.296608	0.996888
49	1	0	5.913037	0.307236	-0.230539

50	1	0	5.521226	-1.219705	0.630031
51	1	0	5.652727	-1.216491	-1.143297

Standard orientation of **3c** at B3LYP/6-31G(d) level in gas phase

Center Number	Atomic Number	Atomic Type	Coordinates (Angstroms) X	Y	Z
1	6	0	-2.537590	3.641202	0.558253
2	6	0	-1.495276	4.550180	0.569756
3	6	0	-0.194595	4.158695	0.268114
4	6	0	-2.300300	2.306874	0.231281
5	6	0	-1.018838	1.904012	-0.071264
6	6	0	0.019027	2.833087	-0.042346
7	7	0	1.220534	2.203508	-0.354658
8	6	0	1.049225	0.852164	-0.429514
9	6	0	-0.436348	0.565412	-0.508364
10	6	0	1.961553	-0.128425	-0.393840
11	6	0	1.481280	-1.562689	-0.445281
12	6	0	0.177908	-1.836382	0.354379
13	6	0	-0.875570	-0.658319	0.318325
14	7	0	-2.144335	-1.009885	-0.329227
15	6	0	-2.243565	-0.518721	-1.702424
16	6	0	-0.901719	0.178128	-1.934734
17	6	0	-3.150320	-1.586407	0.375315
18	8	0	-3.001321	-1.937199	1.517853
19	6	0	-4.465300	-1.787830	-0.346051

20	6	0	0.485004	-2.212649	1.827209
21	6	0	1.290648	-1.203979	2.647625
22	6	0	-0.499462	-3.071748	-0.245921
23	8	0	-0.345083	-3.466469	-1.358013
24	6	0	3.380152	0.221667	-0.294563
25	8	0	3.808422	1.345510	-0.258295
26	8	0	4.182670	-0.831661	-0.236581
27	6	0	5.571978	-0.579543	-0.138992
28	1	0	-3.533303	3.961571	0.804874
29	1	0	-1.687948	5.577491	0.822541
30	1	0	0.615539	4.865081	0.286012
31	1	0	-3.114891	1.606172	0.234902
32	1	0	2.126395	2.584922	-0.193655
33	1	0	1.333125	-1.875486	-1.470359
34	1	0	2.252323	-2.212377	-0.055215
35	1	0	-1.093840	-0.368731	1.332516
36	1	0	-2.395251	-1.330442	-2.402953
37	1	0	-3.068882	0.176323	-1.804569
38	1	0	-0.199312	-0.512652	-2.380373
39	1	0	-0.988211	1.038234	-2.584887
40	1	0	-4.332510	-2.393305	-1.235951
41	1	0	-5.145284	-2.285570	0.328736
42	1	0	-4.895000	-0.839547	-0.651256

43	1	0	-0.459667	-2.399859	2.326517
44	1	0	1.027654	-3.156799	1.825506
45	1	0	1.373700	-1.556730	3.670742
46	1	0	2.294827	-1.077058	2.262037
47	1	0	0.820367	-0.226724	2.679484
48	1	0	-1.165782	-3.604448	0.435634
49	1	0	6.044231	-1.549263	-0.111921
50	1	0	5.917101	-0.018215	-0.994953
51	1	0	5.796368	-0.027769	0.762293

Standard orientation of **3d** at B3LYP/6-31G(d) level in gas phase

Center Number	Atomic Number	Atomic Type	Coordinates (Angstroms) X	Y	Z
1	6	0	-2.514891	3.674948	0.658675
2	6	0	-1.467083	4.569082	0.784163
3	6	0	-0.155355	4.186139	0.519839
4	6	0	-2.271310	2.363681	0.252272
5	6	0	-0.979604	1.971096	-0.014775
6	6	0	0.064012	2.883366	0.127974
7	7	0	1.273289	2.260792	-0.170794
8	6	0	1.094447	0.924311	-0.363698
9	6	0	-0.391359	0.659686	-0.513746
10	6	0	1.990027	-0.072385	-0.414055
11	6	0	1.449696	-1.480838	-0.540192

12	6	0	0.229531	-1.713750	0.385102
13	6	0	-0.890443	-0.622307	0.200986
14	7	0	-2.032492	-1.029522	-0.634086
15	6	0	-2.058006	-0.383474	-1.945972
16	6	0	-0.751915	0.401613	-2.001432
17	6	0	-3.136121	-1.581918	-0.058494
18	8	0	-3.161222	-1.890063	1.104519
19	6	0	-4.335908	-1.815565	-0.952077
20	6	0	0.723251	-1.688906	1.864150
21	6	0	-0.221785	-2.248270	2.935062
22	6	0	-0.342740	-3.094548	0.089372
23	8	0	0.149983	-3.869861	-0.668731
24	6	0	3.412935	0.237677	-0.277494
25	8	0	3.857665	1.346094	-0.126669
26	8	0	4.196616	-0.828725	-0.331849
27	6	0	5.589373	-0.613617	-0.204914
28	1	0	-3.518947	3.988898	0.877976
29	1	0	-1.664359	5.578386	1.098326
30	1	0	0.657303	4.881643	0.626753
31	1	0	-3.089872	1.671435	0.167064
32	1	0	2.178272	2.624367	0.031515
33	1	0	1.179501	-1.716613	-1.563234
34	1	0	2.215511	-2.194767	-0.281790

35	1	0	-1.277262	-0.369172	1.173500
36	1	0	-2.122127	-1.111189	-2.745705
37	1	0	-2.910486	0.280886	-2.030796
38	1	0	0.020164	-0.186313	-2.475792
39	1	0	-0.850995	1.325321	-2.555347
40	1	0	-4.061505	-2.338922	-1.860472
41	1	0	-5.053586	-2.401851	-0.398273
42	1	0	-4.795952	-0.874312	-1.235184
43	1	0	1.658944	-2.237441	1.912146
44	1	0	0.966426	-0.661616	2.113506
45	1	0	0.187332	-2.035049	3.917560
46	1	0	-1.215544	-1.822396	2.885316
47	1	0	-0.324779	-3.325747	2.859978
48	1	0	-1.240954	-3.365405	0.639224
49	1	0	6.044210	-1.589155	-0.279160
50	1	0	5.948523	0.028691	-0.995799
51	1	0	5.819762	-0.164256	0.750232

----

[1] Goto, H.; Osawa, E.; *J. Am. Chem. Soc.* **1989**, *111*, 8950–8951.

[2] Goto, H.; Osawa, E.; *J. Chem. Soc., Perkin Trans. 2*, **1993**, 187–198.

[3] Frisch, M. J.; Trucks, G. W.; Schlegel, H. B.; Scuseria, G. E.; Robb, M. A.; Cheeseman, J. R.; Scalmani, G.; Barone, V.; Mennucci, B.; Petersson, G. A.; Nakatsuji, H.; Caricato, M.; Li, X.; Hratchian, H. P.; Izmaylov, A. F.; Bloino, J.; Zheng, G.; Sonnenberg, J. L.; Hada, M.; Ehara, M.; Toyota, K.; Fukuda, R.; Hasegawa, J.; Ishida, M.;

Nakajima, T.; Honda, Y.; Kitao, O.; Nakai, H.; Vreven, T.; Montgomery, J. A.; Jr., Peralta, J. E.; Ogliaro, F.; Bearpark, M.; Heyd, J. J.; Brothers, E.; Kudin, K. N.; Staroverov, V. N.; Keith, T.; Kobayashi, R.; Normand, J.; Raghavachari, K.; Rendell, A.; Burant, J. C.; Iyengar, S. S.; Tomasi, J.; Cossi, M.; Rega, N.; Millam, J. M.; Klene, M.; Knox, J. E.; Cross, J. B.; Bakken, V.; Adamo, C.; Jaramillo, J.; Gomperts, R.; Stratmann, R. E.; Yazyev, O.; Austin, A. J.; Cammi, R.; Pomelli, C.; Ochterski, J. W.; Martin, R. L.; Morokuma, K.; Zakrzewski, V. G.; Voth, G. A.; Salvador, P.; Dannenberg, J. J.; Dapprich, S.; Daniels, A. D.; Farkas, O.; Foresman, J. B.; Ortiz, J. V.; Cioslowski, J.; and Fox, D. J.; Gaussian *09*, Revision B.01,Gaussian, Inc., Wallingford CT, 2010.

[4]. Bruhn, T.; Hemberger, Y.; Schaumlöffel, A.; Bringmann, G. *Spec Dis*, version 1.60, University of Würzburg, Germany, 2.

Figure S29. Uncropped images of western blot in Figure 7

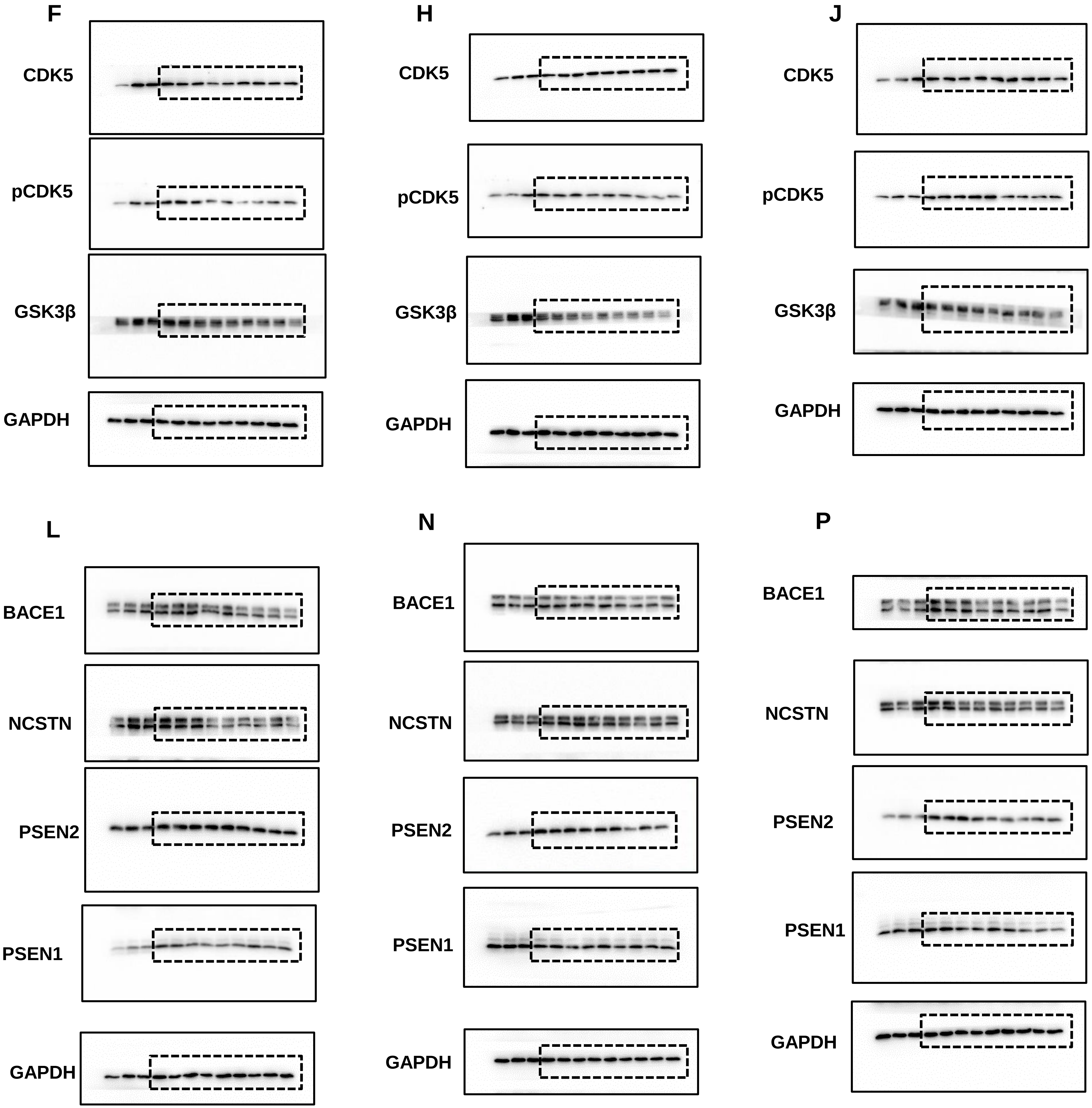

Supplement: Supplementary file 1 [file ijms-24-01487-s001.zip › ijms-2071443-supplementary.pdf]
